# *Arabidopsis NDL-AGB1 modules* Play Role in Abiotic Stress and Hormonal Responses Along with Their Specific Functions

**DOI:** 10.3390/ijms20194736

**Published:** 2019-09-24

**Authors:** Arpana Katiyar, Yashwanti Mudgil

**Affiliations:** Department of Botany, University of Delhi, New Delhi 110007, India; katiyar.arpana@gmail.com

**Keywords:** *Arabidopsis*, *N*-Myc downregulated like, abiotic stress, mannitol, PEG, promoter elements, GUS staining, plant hormones

## Abstract

*Arabidopsis* N-MYC Downregulated Like Proteins (NDLs) are interacting partners of G-Protein core components. Animal homologs of the gene family N-myc downstream regulated gene (NDRG) has been found to be induced during hypoxia, DNA damage, in presence of reducing agent, increased intracellular calcium level and in response to metal ions like nickel and cobalt, which indicates the involvement of the gene family during stress responses. *Arabidopsis* *NDL* gene family contains three homologs *NDL1*, *NDL2* and *NDL3* which share up to 75% identity at protein level. Previous studies on NDL proteins involved detailed characterization of the role of NDL1; roles of other two members were also established in root and shoot development using miRNA knockdown approach. Role of entire family in development has been established but specific functions of NDL2 and NDL3 if any are still unknown. Our *in-silico* analysis of *NDLs* promoters reveled that all three members share some common and some specific transcription factors (TFs) binding sites, hinting towards their common as well as specific functions. Based on promoter elements characteristics, present study was designed to carry out comparative analysis of the *Arabidopsis NDL* family during different stages of plant development, under various abiotic stresses and plant hormonal responses, in order to find out their specific and combined roles in plant growth and development. Developmental analysis using GUS fusion revealed specific localization/expression during different stages of development for all three family members. Stress analysis after treatment with various hormonal and abiotic stresses showed stress and tissue-specific differential expression patterns for all three *NDL* members. All three *NDL* members were collectively showed role in dehydration stress along with specific responses to various treatments. Their specific expression patterns were affected by presence of interacting partner the *Arabidopsis* heterotrimeric G-protein β subunit 1 (AGB1). The present study will improve our understanding of the possible molecular mechanisms of action of the independent *NDL*–*AGB1* modules during stress and hormonal responses. These findings also suggest potential use of this knowledge for crop improvement.

## 1. Introduction

G-proteins are well characterized for signal transduction in response to many environmental stresses like drought, heat, salinity and light intensity. Internalization of AtRGS1 (Regulator of G protein signaling protein) in response to NaCl indicates role of G-protein signaling in stress responses [1]. Expression levels of *AGB1* were significantly upregulated during salt treatment, whereas showed down regulation during heat and cold stress [2]. G-protein interactome has been published using Yeast two hybrid (Y2H) and in planta interaction studies, NDL interactome has also been solved as part of same initiative and hints towards role of NDLs in various stress responses [3]. Animal homolog of plant NDLs consist of four members NDRG1-4. NDRG1 is a ubiquitously expressed intracellular protein that is induced under number of stresses and pathological responses [4]. NDRG1 upregulates under various stress conditions like hypoxia, in the presence of a reducing agent, DNA damage and in response to increased intracellular calcium concentration [5,6,7,8,9]. Various metal ions like nickel, cobalt, and iron are also reported for inducing expression of NDRG1 [10].

Plant NDR proteins were first reported in sunflower (SF21) as pollen, stigma and transmitting tissue cell-specific proteins with putative role, as a signaling molecule in pollen-pistil interaction [11]. Detailed analysis of expression profiles of gene family members in sunflower revealed multiple alternative and organ-specific transcripts indicating spatial and developmental regulation and roles [12,13].

In *Arabidopsis* NDL1 was re-discovered as AGB1/AGG dimmer interacting protein, three homologs had been identified, showing presence of a conserved NDR domain and an alpha/beta hydrolase fold. All NDLs interact with AGB1/AGG (βγ) dimer and C4 domain of RGS1 [14]. Detailed characterization of *Arabidopsis* NDL1 and widespread presence of NDLs from bryophytes to angiosperm hints towards their important functions, though their specific molecular and cellular mechanism of action which still needs to be discovered.

**Figure 1:**
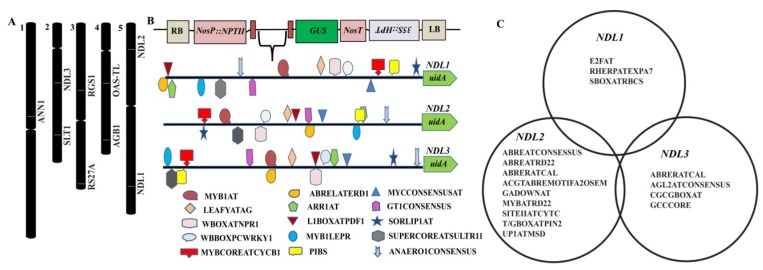
Mapping of the *Arabidopsis* N-MYC Downregulated Like Proteins (*AtNDL)* gene family and putative abiotic stress interacting partners, NDLs stress related interactors (NSRI) on *Arabidopsis* genome along with the stress related elements in the promoter region of *AtNDL* gene family. (**A**) Physical Map of AGI at TAIR using Chromosome Map tool to indicate Chromosomal location of *NDLs* (*NDL1*, *NDL2*, and *NDL3*) and their stress related interactors in *Arabidopsis.* The names of the genes are shown to the right of each chromosome and location of genes is indicating their absolute location on respective chromosome. *RGS1*-Regulator of G protein signaling, *ANN1*-ANNEXIN, *SLTI*-Sodium and Lithium-Tolerant 1, *RS27A*- *Arabidopsis* Ribosomal Protein, *OAS-TL*-O-Acetylserine (Thiol) Lyase. (**B**) In silico analysis of the promoter region of *AtNDL* gene family promoter regions analyzed to get information about common *cis*-regulatory elements present on each of the member. (**C**) Specific *cis*-regulatory elements present on each *AtNDL* member. Different colors are used to indicate for each *cis*-regulatory element.

A recent study involving correlation of the gene expression and morpho-physiological traits under severe water-deficient conditions reports expression of *NDL1* positively correlated with rate of transpiration and projected rosette area [15]. NDLs interactome predicts AtNDL1 interaction with ANNEXIN (drought-responsive gene) Sodium and Lithium-Tolerant 1 (SLT1-salt stress-responsive gene) O-Acetylserine (Thiol) Lyase (OAS-TL) Isoform A1 (characterized for its role during cadmium tolerance) *Arabidopsis* Ribosomal Protein (ARS27A involved in genotoxic stress) hinting us to speculate that AtNDL proteins playing important role in abiotic stress signaling through these NDLs stress related interactors (NSRI), stress signaling downstream components (Figure 1A showing location of all NSRI members on *Arabidopsis* genome) [3].

Previous *NDL* characterization study used miRNA knockdown approach of the entire *NDL* gene family, as well as the detailed overexpression and localization of NDL1, revealed their important role in regulating auxin transport and distribution. Detailed expression/localization profiling of the *NDL1* homologs *NDL2* and *NDL3* had not been done, though all three proteins share 75% identity at amino acid level [14]. Current study aims to dissect out independent functional significance of all three *NDL* gene family members and their role in plant stress management, comparative transcription profiling of all three *NDL* gene family members is performed in silico and *in vivo*. Previously it has been reported that all *NDL* members play role in root as well as shoot development, AGB1 presence is not essential for transcriptional stability of *NDL1* but needed for the stability of NDL1 protein *in planta* [14,16] in order to find-out effect of AGB1 availability on expression profile of *NDL2* and *NDL3* their expression is also studied in *agb1-2* background. The main objectives of the current study are:

1. In silico comparative account of the regulatory elements and expression profiles of *NDL1*, *NDL2,* and *NDL3* to ascertain their characteristics, similarities, and differences.
2. *In planta* comparative analysis of the all three members of the *NDL* gene family during different stages of plant growth and development
3. Comparative expression profile of *NDL* members in response to various abiotic stresses and hormonal treatments in presence and absence of AGB1.

## 2. Results

### 2.1. In Silico Analysis of the Upstream Regulatory Regions of Arabidopsis NDL1, NDL2 and NDL3

Comparative analysis of the *cis*-elements present in the promoter sequences of all three *NDL* members was done using the PLANTPAN 2.0 software. In silico comparative analysis revealed presence of many common TFs binding sites. All three members showed presence of AtMYB2 and AtMYC2 binding sites which are already established characteristic of *NDL* gene family in animals and reason for their nomenclature [17,18]. Abiotic stress-responsive TFs that are involved in dehydration (MYB1AT and ABRELATERD1), sulfur-responsive (SURECOREATSULTR11), phosphate-responsive (PIBS) and SA-induced (WBOXATNPR1), cytokinin-responsive element (ARR1) and defense related TFs like MYB1LEPR binding motif are also present in all the three members of the family. Presence of all these common elements in all three *NDL* genes hints towards the common role of *NDL* family during abiotic and biotic responses. Light-induced TFs like GT1CONSENSUS and SORLIP1AT and developmental and cell cycle-responsive TFs like LEAFYATG and MYBCOREAYCYCB1 are also present in all three members indicating diverse role of *NDL* family members during various stages of plant growth and development (Figure 1B and Table 1).

In addition to these common regulatory elements unique TFs binding sites specific to the promoter region of *NDL1, NDL 2* and *NDL3* are also observed (Figure 1C). *NDL1* promoter showed presence of E2F binding sites indicating its cell cycle-specific functions. Other noted specific elements of *NDL1* promoter region are SBOXATRBCS, important for sugar and abscisic acid (ABA) responses, and RHERPATEXPA7, a root hair specific cis element (Table 2).

*NDL2*, promoter harbors three ABA-responsive elements, ABREATCONSENSUS, ABREATRD22, and ACGTABREMOTIFA2OSEM. It is well established that drought stress triggers production of ABA which in turn induces various other drought inducible genes [19]. Thereby presence of ABA-responsive elements in the promoter sequence of *NDL2* along with MYBATRD22 dehydration-responsive element suggests regulation and involvement of *NDL2* during dehydration/drought stress (Table 2). Along with ABA-responsive elements other specific sites present in the promoter region of *NDL2* are Gibberellic acid (GA) (GADOWNAT) and Jasmonic acid (JA) (T/GBOXATPIN2)-responsive elements. *NDL3* promoter sequence shows presence of one pathogen-responsive element (GCCCORE), and two Ca^2+^/Calmodulin binding sites (CGCGBOXAT) along with AGAMOUS-LIKE2 and HD-ZIP transcription binding sites.

Presence of common TFs binding sites in the upstream promoter regions of all three *NDL* members postulates their involvement in common regulatory mechanisms, while the presence of specific TFs binding sites might attribute unique functions to each *NDL* family member.

### 2.2. Comparative In Silico Expression Analysis of NDL Members

**Figure 2:**
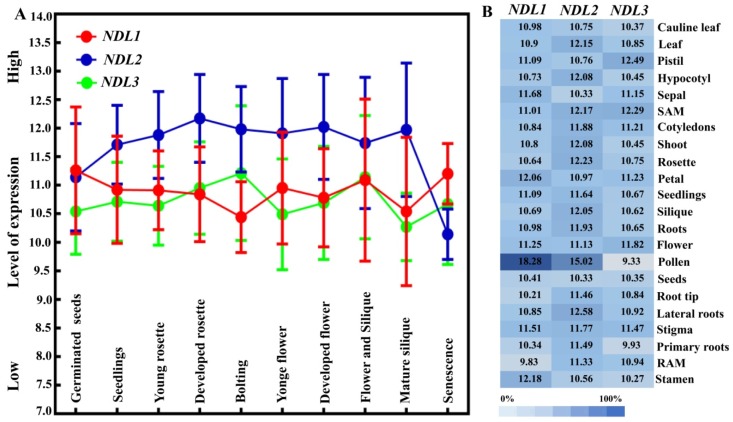
In silico expression analysis of *NDL* gene family. (**A**) GENEVESTIGATOR microarray expression analysis of *NDL1*, *NDL2* and *NDL3* during different developmental stages of plant growth. (**B**) In silico microarray analysis showing tissue-specific expression of *NDL1, NDL2,* and *NDL3* using GENEVESTIGATOR.

Comparative expression analysis of *NDL1, NDL2,* and *NDL3* has been performed using genevestigator microarray data. All three *NDL* members showed expression across all developmental stages, *NDL2* showed expression little higher than *NDL1* and *NDL3* (Figure 2A). Microarray analysis in different plant tissues revealed that *NDL* genes exhibit differential expression pattern across various organs. *NDL1* and *NDL2* showed highest expression in pollen while *NDL3* showed highest expression in carpel and shoot apex (Figure 2B). Since, the role of *NDLs* has been already established in auxin transport during root development [14]. We closely looked at the expression levels of the *NDLs* members in different parts of roots and found that expression level of all the members were comparable, in primary roots *NDL1* and *NDL2* is comparatively little higher than *NDL3*. Overall expression pattern revealed ubiquitous expression of the gene family which indicates important function of the family members during plant growth and developmental responses (Figure 2B).

### 2.3. Comparative In Vivo Expression Analysis of NDLs during Early Stages of the Plant Growth

**Figure 3:**
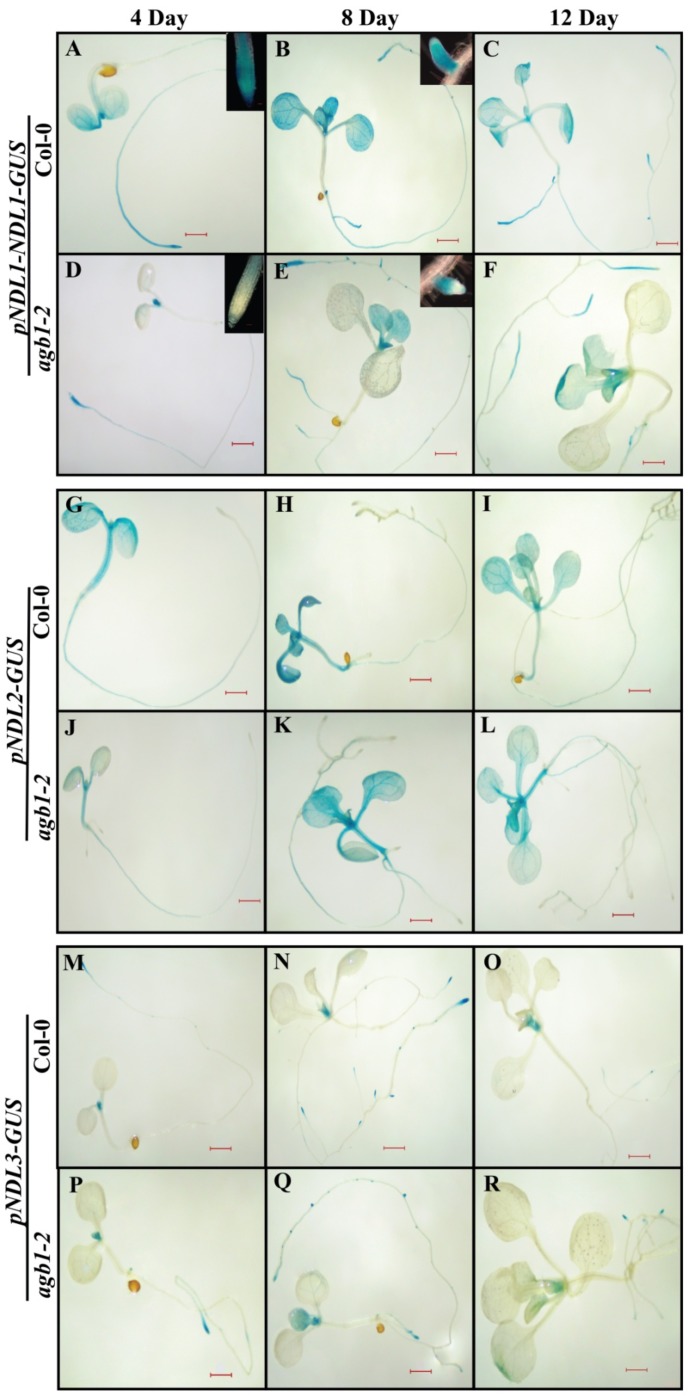
*In planta* expression analysis of *NDL* gene family during different developmental stagesin presence and absence of *AGB1*: (**A**–**C**, **G**–**I** and **M**–**O**). Histochemical GUS staining of *pNDL1-NDL1-GUS*, *pNDL2-GUS* and *pNDL3-GUS* seedlings in wild type Col-0 background. (**D**–**F**, **J**–**L** and **P**–**R**) Histochemical GUS staining of *pNDL1-NDL1-GUS*, *pNDL2-GUS* and *pNDL3-GUS* seedlings in *agb1-2* mutant background. Histochemical GUS analysis in transgenic *Arabidopsis* plants during different stages of development was carried out 4 day, 8 day, and 12 day old seedlings were analyzed. GUS expression is under the control of *NDL2* and *NDL3* promoter and translational fusion in case of *NDL1*, Scale Bar = 0.2 μM.

*In-vivo* expression analysis of all three *NDL* members showed that the localization pattern of GUS is quite unique for each member. During the initial stages of plant growth (4–12 day-old seedlings) *pNDL1-NDL1-GUS* showed localization in cotyledonary leaves, true leaves, various parts of primary and lateral roots including elongation zone and root apical meristem (RAM) (Figure 3A–C and Table 3). *pNDL2-GUS* showed GUS expression in cotyledonary leaves, true leaves, hypocotyl and maturation zone of primary root, but strikingly missing from root tip and in lateral roots (Figure 3G–I and Table 3). *pNDL3-GUS* expression was missing in cotyledonary leaves, present in newly emerging true leaves, present in the RAM of the primary root and in lateral roots (Figure 3M–O and Table 3).

### 2.4. Comparative In Vivo Expression Analysis of NDLs in Absence of AGB1

Previously, we have found that presence of AGB1 doesn’t affect transcriptional levels of *NDL1* but necessary to maintain the post translational steady state level of NDL1 [14]. Since all three NDL members had shown physical interaction with AGB1 in Y2H we hypothesized that NDL2 and NDL3 might require its partner, AGB1, either to regulate expression levels or for post-translational stability similar to NDL1. In order to test this speculation, expression of *pNDL2-GUS* and *pNDL3-GUS* was studied in *agb1-2* background.

In *agb1-2* background *pNDL1-NDL1-GUS* localization was absent from cotyledonary leaves (Figure 3D–F and Table 3) but present in true leaves. In case of roots the localization was absent from both primary and lateral roots RAM (Figure 3D,E inset) whereas present in other parts of roots similar to Col-0 background. In case of *pNDL2-GUS* and *pNDL3-GUS* localization pattern of the GUS was same in *agb1-2* mutant background as compared to wild type Col-0. (Figure 3J–L for *NDL2*, Figure 3P–R for *NDL3* and Table 3). These finding indicate that expression levels of both *NDL2* and *NDL3* are also unaffected by the absence of *AGB1* similar to previously reported for *NDL1.*

### 2.5. In Silico Expression Analysis of NDLs under Various Stress and Hormonal Treatments

Previous study has confirmed that *NDLs* play role in auxin transport during lateral root formation [14]. Correlation of gene expression and responses of morpho-physiological traits under severe water-deficient conditions indicate substantial alteration in expression levels of *AtNDL1* during drought stress responses using tanscriptomic analysis [15]. In order to find out if independent NDL members play independent specific roles or have combined redundant functions during abiotic stress exposure and hormonal treatments, we have analyzed their comparative expression profiles from publically available databases. (Genevestigator and eFP browser).

**Figure 4:**
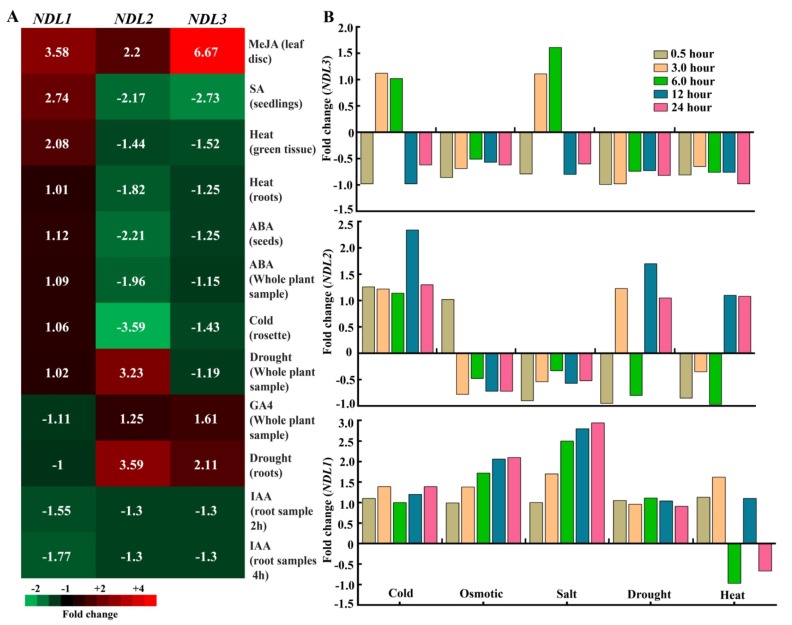
In silico expression analysis of *NDL* gene family during abiotic stress and hormonal treatment using genevestigator and eFP browser: (**A**). Comparative *in-silico* expression analysis of *NDL1, NDL2,* and *NDL3* using GENEVESTIGATOR microarray data, under different abiotic stress and hormonal treatments (**B**). In silico analysis of *NDL1, NDL2,* and *NDL3* during abiotic stress treatments at different time points (0.5, 3, 6, 12, and 24 h) comparative expression is shown as fold change compared to no stress control.

Genevestigator Microarray data revealed that after auxin (IAA) treatment, expression levels of *NDL1* decreased to nearly 1.77 fold, expression of *NDL2* and *NDL3* showed a similar trend, decrease of around 1.3 fold. (Figure 4A). In presence of ABA, *NDL2* showed a decrease (2.21 fold) in expression level, as compared to *NDL1* and *NDL3* which showed an upregulation up to ~1.12 fold and down regulation of nearly 1.25 fold respectively. Unlike *NDL2* and *NDL3*, *NDL1* showed slight decrease (1.1 fold) in its expression in response to GA (Figure 4A).

All *NDL* members also showed a response toward various abiotic stresses (heat, cold and drought). In case of heat treatment in green tissue *NDL1* showed increase (2.08 fold) as compared to *NDL2* and *NDL3* which showed a decrease in expression (1.44 and 1.52 fold respectively) in case of roots also similar trend was there. Even for cold treatment *NDL1* showed positive regulation (1.06 fold up) while down regulation was observed for *NDL2* and *NDL3* (3.59 and 1.43 fold respectively). Under drought stress all members have positive response, *NDL2* showed upregulation in expression (3.59 fold) followed by *NDL3* and *NDL1* (increase of 2.11 and 1.60 fold respectively, Figure 4A). The observed high expression of *NDL2* gene in drought stress thereby could possibly be linked to the fact that multiple ABA-responsive factor binding elements are present in promoter sequence of *NDL2*.

To find out optimal time point for the in vivo expression analysis we also performed comparative in silico expression analysis of *NDLs* at different time points during various abiotic stress treatments using eFP browser. For *NDL1* maximum transcript levels compared to no-treatment control were detected at 24 h. In case of *NDL2* expression, levels fluctuated during different stress treatments and variation was found at different time points but overall maximum levels were observed at 24 h. A trend similar to *NDL2* was observed for *NDL3* expression during different treatments and time points (Figure 4B)

### 2.6. In Vivo Expression Analysis under Abiotic Stresses and Hormonal Treatments

In order to find out maximum expression levels for all three *NDLs* promoter GUS/β-glucuronidase activity was tested using 4-Methylumbelliferyl-β-D-glucuronide (MUG) assay for two time points (6 h and 24 h) in Col-0 background, no significant difference was found between expression levels of *NDLs* and two time points. (Supplementary Figure S1). Based on the in vivo GUS/β-glucuronidase activity levels and in silico analysis of the expression profiles of all three *NDL* members, treatment with various hormones and abiotic stresses were designed. Six-day-old seedlings were subjected to 24 h treatment with various hormones (ABA, GA, IAA, JA, SA, and BAP) and abiotic stresses (cold, heat, Mannitol, PEG and salt) followed by measurement of GUS activity either histochemically or by fluorometry.

**Figure 5:**
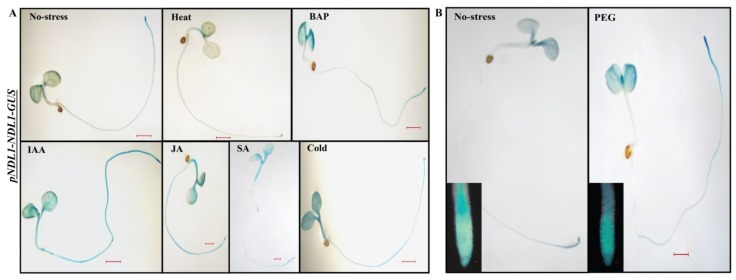
In vivo histochemical GUS activity for *NDL1* in wild type background in response to different abiotic stresses and hormones: (**A**). Histochemical analysis of GUS activity for *NDL1* in wild type background in response to different abiotic stress and hormones. Changes in intensity of GUS staining were detected with Heat, Cold, IA, JA, SA and BAP. GUS staining was done in seedlings that were six days old and 24 h of stress treatment followed. (**B**) Histochemical GUS staining of 6-D-old *pNDL1-NDL1-GUS* seedlings in wild type Col-0 background. Increased levels of GUS staining intensity detected with PEG at root tip (inset). Result shown is representative of three independent biological replicates (*n* ≥ 10 in each experiment). Scale Bar = 0.2 μM for seedlings and 0.02 µM for inset RAM.

*pNDL1-NDL1-GUS*, showed increase in the GUS staining intensity when treated with cold, IAA (auxin), JA, Salicyclic acid (SA), BAP (cytokinin), and Poly ethylene glycol (PEG) and almost no staining observed with heat treatment (Figure 5). GUS staining remained unaffected after treatment with ABA (Supplementary Figure S2), GA (Supplementary Figure S3) and mannitol (Supplementary Figure S4). In case of salt treatment, no difference was found in root but hypocotyle showed increase in the GUS staining intensity (Supplementary Figure S5) compared to no stress treatment control.

**Figure 6:**
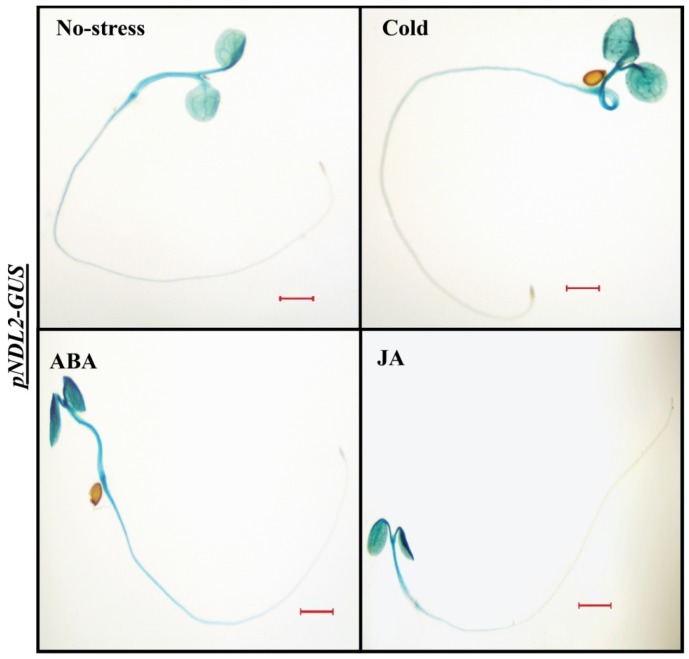
In vivo histochemical analysis of GUS activity for *NDL2* in wild type Col-0 background in response to different abiotic stresses and hormones: Histochemical GUS staining of 6-D-old transgenic *Arabidopsis* seedlings (*pNDL2-GUS)* in wild type Col-0 background. Increased intensity levels of GUS staining detected with ABA, cold, and JA. Result shown is representative of three independent biological replicates (*n* ≥ 10 in each experiment). Scale Bar = 0.2 μM.

*pNDL2-GUS* showed increased levels of GUS staining in the cotyledons and hypocotyl after treatment with ABA, cold and JA (Figure 6), primary root showed no expression similar to no treatment control. BAP (Supplementary Figure S2), IAA, GA, heat (Supplementary Figure S3), Mannitol and PEG (Supplementary Figure S4) and SA (Supplementary Figure S5) and other treatments had no effect on expression level as no difference was observed compared to no stress treatment control.

**Figure 7:**
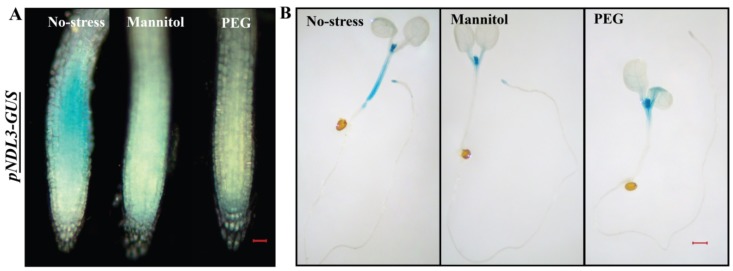
In vivo histochemical analysis of GUS activity for *NDL3* in wild type Col-0 background in response to different abiotic stresses and hormones: (**A**). Histochemical GUS staining of 6-D-old transgenic *Arabidopsis* seedlings (*pNDL3-GUS)* in wild type Col-0 background. Changes in intensity of GUS staining detected with PEG and Mannitol in RAM. (**B**) and in hypocotyl part of the seedlings. Result shown is representative of three independent biological replicates (*n* ≥ 10 in each experiment). Scale Bar = 0.2μM for seedlings and 0.02 μM for RAM.

In case of *pNDL3-GUS,* no difference in the GUS staining was detected when seedlings were treated with ABA, BAP, cold (Supplementary Figure S2), IAA, GA, JA, heat (Supplementary Figure S3), salt and SA (Supplementary Figure S5). Mannitol and PEG treatment resulted in decreased level of *pNDL3-GUS* expression in RAM and hypocotyl in comparison to no treatment control which showed diminished staining at both the places. GUS staining was absent in the rest of the seedlings, same as in case of no treatment control of six-day old seedling (Figure 7 and Supplementary Figure S4).

Results indicate that all the three family members’ show independent response when encountering various stress/hormonal treatments. Both *NDL1* and *NDL3* after treatment with Mannitol and PEG showed difference in GUS intensity indicates that both the members are working when plants encounter with drought and osmotic stress. Although both the members have altered expression in same stress mechanism of functioning seems opposite as *NDL1* showed up regulation after PEG treatment while *NDL3* showed down regulation when treated with PEG (compare Figure 5B and Figure 7), while *NDL2* expression remains unaffected by both treatments in tested plant stage (Supplementary Figure S4).

### 2.7. In Vivo Expression Analysis of NDLs in Absence of AGB1 under Abiotic Stress and Hormonal Treatments

Previous study has reported that expression levels of *NDL1* were not affected but protein steady state levels were affected by AGB1 [14]. In order to find out role of AGB1 on expression levels of *NDL2* and *NDL3* their expression patterns were studied in *agb1-2* mutant. We found that expression of *NDL2* and *NDL3* were not affected in *agb1-2* mutant and stays same as in wild type Col-0 background (Figure 3). In order to find-out whether AGB1 has any role on expression during abiotic stress or hormonal treatments expression pattern was observed for all the three members in absence of AGB1 after abiotic (cold, heat, Mannitol, salt and PEG), and hormonal (ABA, GA, IAA, JA, SA and BAP) treatments.

**Figure 8:**
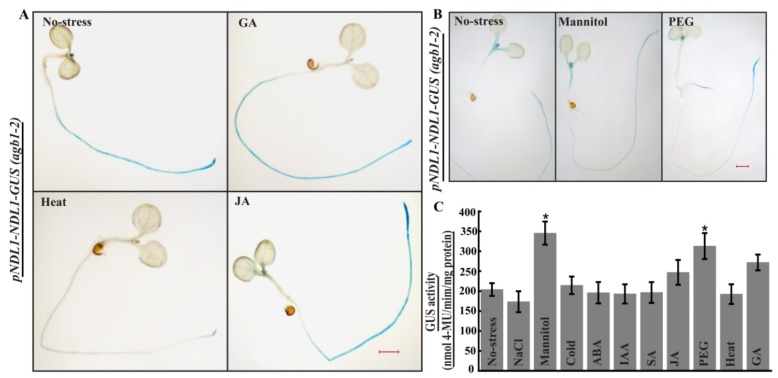
In vivo histochemical GUS staining of 6-day old transgenic *Arabidopsis* seedlings (*pNDL1-NDL1-GUS*) in *agb1-2* mutant background: Histochemical GUS staining was done in transgenic *Arabidopsis* seedlings that were six days old treatment was given for 24 h. (**A**) Changes in GUS intensity levels were detected with GA, heat and JA. (**B**). Increased GUS staining detected for Mannitol and PEG at the cell division and elongation part of the primary root. (**C**) Abiotic stress treatments followed by fluorometric MUG assay was done for quantitative estimation of stress response. Significantly higher GUS activity was obtained for Mannitol and PEG treatment compared to No-stress control indicative of NDL1 involvement during osmotic and drought responses (Students *t*-test = * *p* value < 0.05, Error bars represent SD). Result shown is representative of three independent biological replicates (*n* ≥ 10 in each experiment). Scale Bar = 0.2 μM.

*pNDL1-NDL1-GUS* showed increased GUS intensity in primary root of the seedlings compared to no treatment control for GA, Mannitol, PEG and JA while no staining was detected in the primary root after heat treatment (Figure 8A,B). MUG assay was also performed and significantly increased GUS activity was observed only for PEG and Mannitol treatment in comparison to no-treatment control seedlings (Figure 8C). GUS staining intensity remains unaffected for ABA, BAP, cold (Supplementary Figure S2), IAA (Supplementary Figure S3), salt and SA (Supplementary Figure S5) treatments. Response of ABA and salt for *pNDL1-NDL1-GUS* remained similar in both the background (in wild type Col-0 as well as in *agb1-2* mutant) meaning presence of AGB1 don’t make any difference on expression, while for cold, IAA, SA and BAP upregulation was detected in the cotyledons and in primary root in comparison to no treatment control seedlings in case of wild type

Col-0 background while remains unaffected in *agb1-2* mutant background meaning presence of AGB1 is vital for NDL1 stability during cold, IAA, SA and BAP treatment, indicating stress specific role of NDL1-AGB1 module.

The expression analysis for *pNDL2-GUS* in terms of GUS staining was also observed in absence of AGB1. Seedlings were treated with ABA, cold, IAA, GA, heat, JA, Mannitol, PEG, salt, SA and BAP. No difference in GUS staining was detected in *agb1-2* mutant background compared to no treatment control (Supplementary Figures S2–S5) suggesting no role/requirement of AGB1 for *NDL2* expression during treatment in *agb1-2* background. In case of cold, ABA and JA treatment upregulation of *pNDL2-GUS* was detected in the cotyledons in wild type Col-0 background (Figure 6) but in *agb1-2* background this effect was not present (Supplementary Figures S2–S5) meaning AGB1 presence is required during cold, ABA and JA treatments for *NDL2* expression upregulation.

**Figure 9:**
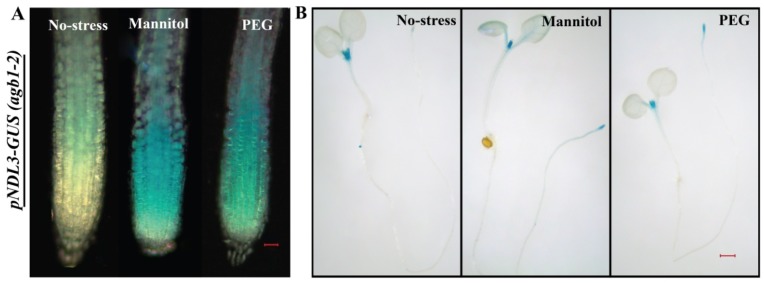
In vivo histochemical GUS staining of 6-day old transgenic *Arabidopsis* seedlings (*pNDL3-GUS*) in *agb1-2* mutant background: (**A**). Increased levels of GUS staining intensity were detected in RAM compare to almost no staining in no treatment control with Mannitol and PEG treatment. (**B**) Histochemical GUS staining was analysed in whole seedling also. ne Six days old *Arabidopsis* transgenic seedlings were subjected to treatment for 24 h. Result shown is representative of three independent biological replicates (*n* ≥ 10 in each experiment). Scale Bar = 0.2 μM for seedlings and 0.02 μM for RAM.

In case of *pNDL3-GUS*, no difference in expression was detected in for ABA, BAP, Cold (Supplementary Figure S2), IAA, GA, JA, heat (Supplementary Figure S3), salt, SA (Supplementary Figure S5) in comparison to no treatment control. For all these treatments results were similar in both the background in wild type as well as in *agb1-2* mutant background. Very interestingly after treatment with PEG and Mannitol in *agb1-2* mutant background intensity of GUS staining showed increase in the RAM which is opposite effect compared to the Col-0 background where a decline in hypocotyl and almost absence of expression was observed in RAM (Figure 7). This means differential and opposite role of AGB1 is operating for regulating *NDL3* expression under normal and dehydration stress condition (Figure 9). Presence of AGB1 is needed under stress condition to downregulate *NDL3* expression, which might need to be downregulated under dehydration stress.

## 3. Discussion

Comparative analysis for all three members of *NDL* gene family revealed their role in many common and unique functions. *In-silico* analysis revealed that all three members share many common transcription factor binding sites (Table 1). Presence of MYC binding factors postulate evolutionary conserved regulatory mechanisms of downregulation similar to animal systems for *AtNDL* family. Regulatory regions of all the three members contain motifs for stresses (biotic and abiotic), development, and for hormonal responses. MYB recognition sites like MYB1AT and MYB1LEPR are present in all the *NDL* members. MYB1AT is a MYB recognition site which is also present in the promoter of *RD22* which is ABA-induced and drought-responsive gene in *Arabidopsis* [18]. Another dehydration-responsive motif ABRELATERD1 is also present in the *NDL* family members, this motif has been reported in the up-stream region of the *ERD1* (early response to dehydration 1). Promoters with *ERD1* motif are involved in response to ABA and also show significant upregulation under water stress [20]. Both AtMYC and AtMYB are known to function as transcriptional activators in abscisic acid signaling presence of these motifs in combination with dehydration-responsive motifs in *NDL* family members indicates their role in ABA-dependent early response to dehydration.

In the present study, we have only analysed early stage of plant growth but Rymaszewski et al., 2017 has analysed plant growth starting from early stages till reproductive stage. They found positive co-relation of morpho-physiological traits like projected rosette area, and increased transpiration rate under low water-deficient conditions and expression levels for *NDL1*. They also speculate that these acclimations may be driven by largely stress hormone ABA. In another study Du et al., 2018 [21] has also found that during low water treatment conditions plants shifts for drought escape mechanism and leads to early flowering to complete their life cycle. Activation of the flowering genes are both ABA-dependent and ABA-independent [21]. All these finding indicates the complex network between stress, hormones and developmental responses.

Interestingly both AGB1 and GPA1 both are interacting partners of NDL members [14] are also interacting partners of one of the MYB (AT3G24120) in G protein interactome using in Y2H [3].

ANAERO1CONSENSUS motif is indicative of involvement of the gene family during anaerobic conditions. MYCCONSENSUSAT regulates transcription of *CBF/DREB1* genes during cold. Presence MYC recognition sites had been postulated to regulate the gene transcription by a basic bHLH transcription activator during cold stress [22]. MYCCONSENSUSAT sites are present mainly at the genes which have more expression in seed and the response to cold is ABA-mediated [23]. MYCCONSENSUSAT motif was also detected in the promoter of the gene which showed response for JA. WBOXATNPR1 is a disease related motif that is established as a site for binding SA-induced WRKY TFs [23]. Presence of a P1BS and SURECOREATSULTR11 motif indicative of the involvement of the family members for nutrient availability. P1BS motifs are associated with phosphate starvation response, while SURECOREATSULTR11 are found in the genes involved during sulfur deficiency responses (*SULTR1; 1*) [24,25].

All the three *NDL* members contain various hormone-responsive transcription factors binding sites. Responses of MYCCONSENSUSAT and ABRELATERD1 are ABA-dependent during desiccation and cold responses. MYCCONSENSUSAT motif also present in the genes which are involved in JA-mediated defense responses. Cytokinin response regulator ARR1AT and SA-responsive WBOXATNPR1 is also present commonly in all the three family members. Light-responsive motifs GT1CONSENSUS and SORLIP1AT are also common in all the members. GT1CONSENSUS a light-responsive motif, also showed tissue-specific localization, it is present in the up-stream region of the right regulated genes which are highly expressed in leaf [23]. Other than these common shared regulatory elements unique TFs binding sites to *NDL1*, *NDL2* and *NDL3* were also observed (Table 2). *NDL1* showed presence of exclusive binding sites for E2F, which targets cell cycle-regulated expression. E2F controls cell cycle by the regulation of the transcription of the genes that are involved during cell cycle and DNA replication [26,27] *Arabidopsis* E2F are classified as classical E2F proteins (E2Fa-c) and atypical E2F proteins (E2Fd-f) [28]. In G-protein interactome database NDL1 shows interaction with CKS2 (cell cycle regulatory subunit) and cyclin-dependent kinase G1 hinting specific function during cell cycle. Previously it is established that sucrose and D-glucose enhances the stability of NDL1 protein [14] which goes in accordance with presence of SBOXATRBCS, important for sugar and ABA responses. Role of NDL1 during primary and lateral root growth and development is already established presence of RHERPATEXPA7, a root hair specific cis-element needs further probing to study role of NDL1 in root hair development.

Likewise, *NDL2* strikingly shows presence of various kinds of ABA-responsive elements like ABREATCONSENSUS, which play role during ABA signaling and responsible for abiotic stress tolerance [29], ABREATRD22 and ACGTABREMOTIFA2OSEM. ACGTABREMOTIFA2OSEM motif present in the promoter of rice *OsEm* gene, which is regulated by seed specific transcription factor and ABA-responsive [30]. ACGTABREMOTIFA2OSEM motif also found in maturing seeds and acts as a binding site for bZIP TFs ABI5 [31]. Those genes which are highly expressed in mature seeds of *Arabidopsis* are found to be enriched with ABRE motif [32]. A single copy of ABRE is not sufficient for ABA-mediated responses. Multiple copy number of ABRE along with a coupling element forms the active complex for ABA-mediated responses [33]. Along with ABA-responsive binding sites several other sites like, GA and JA-responsive sites GADOWNAT and T/GBOXATPIN2 are also present in the promoter region of *NDL2*. GADOWNAT is common sequence found in genes which downregulates after GA treatments. GADOWNAT is shown to be identical as ABRE [34], also hints the regulation of gene under ABA responses. T/GBOXATPIN2 is a wounding response motif, found in the promoter of JA-responsive genes [35]. As per the *in-silico* analysis presence of different kinds of ABA response factors and seed specific transcription factors which are again ABA-dependent for their responses. Since our in vivo expression analysis proved absence of the *NDL2* expression in the roots. *NDL2* members might be specifically involved during seed germination and growth responses.

The *NDL3* promoter showed presence of -ABRERATCAL, an early stress-induced motif related to ABRE and found in the up-stream region of Ca^2+^ ion-responsive genes, CGCGBOXAT is a Ca^2+^-dependent Calmodulin binding motif is also present both indicative of involvement of *NDL3* during calcium and ABA-mediated stress adaptations [36,37,38]. GCCCORE has been found in the promoter region of the several pathogen-responsive genes showed JA-dependent defense responses [39]. HDZIPIIIAT involved in vascular differentiation and patterning [40] and required for polar auxin transport in shoot [41], *NDLs* combined role is already established for polar auxin transport in roots [14] Increased HDZIPIIIAT activity leads to formation of extra cotyledons [42], a phenotype somewhat similar to tricot phenotype of *ndl* microRNA downregulated lines [16]. While downregulation of HDZIPIIIAT showed the loss of cotyledons formation [42,43]. All these findings indicate that *NDL3* might be a Ca^2+^-responsive gene during stress signaling. *NDL3* also involved in similar functions like *NDL1* as both share similar in vivo localization during early stages of plant growth

The combinatorial effect of unique and common transcription factors present in *NDL* gene family essentially suggests that *NDL1, NDL2,* and *NDL3* do share a basic common regulatory mechanism of downregulation by MYC/MYB. Our in vivo analysis also shows that during different stages of life cycle, and when plant encounter specific stress conditions differential regulation of all *NDL* members is possible and this could be attributed to the unique transcription factor binding sites present in each gene respectively.

Initial developmental study for all three members of the family showed that they are very specific about their expression pattern. In wild type background, *NDL1* was expressed in cotyledonary leaves, true leaves, primary and lateral roots. Functional characterization of *NDL1* has been done in detail and it has been found that *NDL1* is required for primary root and shoot meristem initiation growth and lateral root/shoot branching [14,16]. For *NDL2* the expression was detected in cotyledonary leaves, true leaves and in maturation zone of primary root, no expression was detected in RAM and in lateral roots (Figure 3) indicating function in different organs compared to *NDL1* and *NDL3*, which share overlapping expression zones. Presence of GA and cold-responsive elements, along with different kind of ABA-responsive factors in *NDL2* promoter is suggestive of its involvement during seed germination and early growth as during low temperature ABA biosynthesis and GA catabolism is up regulated that lead to seed dormancy [44].

*In silico* and in vivo expression analysis in case of *NDL3* was detected in newly emerging true leaves and at primary and lateral root tips (Figure 3). *NDL3* show response during drought stress and expression pattern is again quite similar with *NDL1* response (Figure 7; Figure 9) meaning, both the members share overlapping expression patterns in vivo and are playing role in similar physiological processes.

Previously it has been found that in *agb1* mutant *NDL1* expression levels are of wild type level meaning *NDL1* transcript is unaffected by the AGB1 (Figure 5D) [14] Similar to *NDL1* study we found that in vivo expression levels/patterns of *NDL2* and *NDL3* remains similar to Col-0 levels even in *agb1-2* mutant, meaning AGB1 does not affect the transcript levels of *NDLs.*

NDL1 protein stability in young primary root needs presence of AGB1, as NDL1 undergoes proteosomal degradation in absence of AGB1 [14], effect of AGB1 on stability and localization of NDL2 and NDL3 is still pending and needs to be characterized.

The effect of different hormones and abiotic stress responses also shows that along with the common response of *NDL1* and *NDL3* during osmotic/drought stress the different family members show specific responses for various treatments. NDL1 localization is upregulated during cold, IAA, JA, SA and cytokinin treatment and downregulated after heat stress (Figure 5A), meaning presence of AGB1 is essential for NDL1 stability during cold, IAA, JA, SA and BAP treatment, indicating stress specific role of NDL1-AGB1 module.

Although *NDL1* and *NDL3* share similar expression domains, *NDL3* expression remains unaffected by cold, IAA, JA, SA, cytokinin and heat treatments (Supplementary Figures S2–S5) meaning differential specificity in their functions.

Various ABA binding sites are present in the promoter region of *NDL2* and in accordance after ABA treatment (also for cold and JA) the *NDL2* expression shows upregulation in Col-0 background but did not showed any difference in *agb1-2* mutant background indicating presence of AGB1 is essential for *NDL2* expression upregulation during these treatments.

In case of *NDL3* expression upon treatment with Mannitol and dehydration stress it was found that AGB1 is negatively regulating *NDL3* expression under normal and dehydration stress condition (Figure 9). Presence of AGB1 is needed under stress condition to downregulate *NDL3* expression, which may be downregulated under dehydration stress.

Although various common TFs have been found in the promoter analysis but all the three members did not show the response for each of them. Every members of the family behaves differentially even though they share the common regulatory motifs. This happens because the family members also showed differential expression pattern and response to various stresses and hormones could be specific for particular developmental stage, our analysis is limited for initial growth stage of the plant. Previously it was found that AGB1 protein is needed for NDL1 stability and here we also found that the protein stability was affected in mutant background. In addition, *AGB1* was also confirmed to be involved in drought stress [45]. *NDL1* is also predicted to be a stress marker for drought [15]. Our *in-vivo* study proves that during the combined function out of three members two of them (*NDL1* and *NDL3*) are involved during drought stress response. *NDL1* is acting as a general abiotic stress responder during heat, cold, IAA, JA, SA and in cytokinin responses as protein steady state levels show upregulation after all these treatments, while *NDL3* expression remains unaffected by all these treatments, meaning limited and specific role. *NDL2* doesn’t show any alteration in expression after Mannitol and PEG treatments but showed significant upregulation for ABA, cold, and JA treatment.

In summary dehydration stress (Mannitol and PEG treatments) caused increase in steady state protein levels of NDL1 in both wild type Col-0 as well as in *agb1-2* mutant background. Whereas *NDL3* showed downregulation of expression in wild type Col-0 background while upregulation in *agb1-2* mutant background in RAM. Expression levels of *pNDL2-GUS* were affected in AGB1-dependent manner during cold, ABA and JA treatment. These results strongly support the direct involvement of *NDL1* and *NDL3* during osmotic/drought stress responses and *NDL2* might be playing ABA-dependent indirect role. AGB1 is also playing role in differential regulation of expression of these *NDLs* members during different treatments by affecting protein stability (seen in case of NDL1) or transcript levels (as seen in case of *NDL3* expression) in stress specific manner.

## 4. Material and Methods

### 4.1. Plant Material and Growth Conditions

*Arabidopsis* Col-0 ecotype was used in the present study. *Agrobacterium* (strain GV3101)-mediated floral dip method [46] was used to generate transcriptional fusion transgenic (*pNDL2, 3-GUS*) in wild type Col-0 as well as in the *agb1-2* mutant background. *pNDL1-NDL1-GUS* translational fusion lines were taken from previous study [14]. Transformed seeds were selected on ½ MS medium (Himedia, Mumbai, India) containing 25 mg/L Hygromycin (Duchefa, Amsterdam, Netherland), resistant plants were moved to the soil and grown to maturity in a growth room with a photoperiod 16h light/8h dark at 22 °C, and the light intensity of 100 μmolm^−2^s^−1^. Three independent single insertion T3 homozygous lines were obtained and used for developmental and stress treatments. For developmental study, seeds were grown vertically for, 4-day, 8-day and 12-day respectively followed by in vivo GUS assay. For stress treatment and fluorometric analysis six day old seedlings were used.

### 4.2. Isolation and Cloning of NDL2 and NDL3 Promoters

Genomic DNA was isolated with minor modifications from the *Arabidopsis* thaliana Col-0 using Doyle method [47]. Primers listed in Supplementary Table S1 were used to amplify the promoter region of *NDL2* and *NDL3*. Amplified fragments were then cloned into a pENTR/D-TOPO entry vector (Invitrogen, Massachusetts, United States) gateway, followed by pGWB3 destination vectors) with C-terminus GUS reporter. For NDL1 previously published lines were used [14].

### 4.3. In-Silico Analysis

Sequence information and location of *NDL1, NDL2* and *NDL3* was retrieved from https://www.arabidopsis.org/. In silico analysis was done using online programs PLANTPAN 2.0 [48]. For the expression pattern of *NDL* genes in different stages of development in different tissues, and time points data from the free version of the GENEVESTIGATOR online portal (https://www.genevestigator.com/gv/plant.jsp) and eFP browser (http://bar.utoronto.ca/efp/cgi-bin/efpWeb.cgi) was used for analysis.

### 4.4. GUS Staining Assay

Three independent lines for *pNDL1-NDL1-GUS* and published *pNDL1-NDL1-GUS* lines were germinated and grown on ½ MS media for 4-day, 8-day and 12-days. Gus staining on the seedlings was done using Jefferson method [49].

### 4.5. Fluorometric GUS Assay

For quantitatively GUS activity. 0.5 g of seedlings (6-day old) were harvested and frozen into liquid N_2_ in 1.5 mL micro centrifuge tube, followed by grinding and protein extraction in extraction buffer (50 mM sodium phosphate buffer, pH 7.0, 10 mM EDTA, 0.1% Triton *X*-100, and 10 mM β-mercaptoethanol). The homogenate was centrifuged at 10,000 g for 20 min at 4 °C. Supernatant was collected and assayed for protein concentration using Bradford method [50]. Five microgram of protein was added to GUS assay buffer (1*X* = 50 mM sodium phosphate buffer, pH 7.0, 10 mM EDTA, 0.1% Triton *X*-100, and 10 mM β-mercaptoethanol containing 2 mM MUG (Himedia, Mumbai, India) total volume was made up to 500 μL using ddH_2_O. Samples were incubated at 37 °C for one hour followed termination of the reaction using 400 μL of 0.2 M Na_2_CO_3_. For quantitative GUS analysis duplicate samples were assayed. The reaction product 4-methylumbelliferon (MU) was detected fluorometrically at excitation and emission of 365 nm and 455 nm respectively [51] using Tecan Spark multimode micro plate reader. GUS activity was expressed in nmol MU min^−1^ μg^−1^ protein.

### 4.6. Hormone and Abiotic Stress Treatments

Seeds were stratified and grown vertically on ½ MS media. Six day old seedlings were subjected to 24 h treatment of sodium chloride (NaCl; 150 mM), Mannitol (300 mM), cold (4 °C), heat (37 °C), abscisic acid (ABA; 20 μM), Indole-3-acetic acid (IAA; 10 μM), salicylic acid (SA; 10 μM), methyl jasmonate (JA; 10 μM), polyethylene glycol (PEG 6000, 20%) and Gibberellic acid (GA; 20 μM) in liquid MS. NaCl, Mannitol and PEG were obtained from Himedia (Himedia, Mumbai, India) all the other hormones and fine chemicals were obtained from Sigma (Sigma, Missouri, United States).

### 4.7. Accession Numbers

Sequence data from the article can be found from https://www.arabidopsis.org/using accession number: AT5G56750 (*NDL1*), AT5G11790 (*NDL2*), AT2G19620 (*NDL3*) and AT4G34460 (*AGB1*). Chromosomal locations of the respective genes are as follows: NDL1: Chromosome 5 (22957629-22960916), NDL2: Chromosome 5 (3799408-3803216), NDL3: Chromosome 2 (8485991-8488963), AGB1: Chromosome 4 (16477031-16479620), Regulator of G protein signaling protein-RGS1: Chromosome 3 (9532613-9535629), ANNEXIN-ANN1: Chromosome 1 (13225168-13227239), Sodium and Lithium-Tolerant 1-SLTI: Chromosome 2 (15761295-15763451), *Arabidopsis* Ribosomal Protein -RS27A: Chromosome 3 (22611521-22612905), O-Acetylserine (Thiol) Lyase -OAS-TL: Chromosome 4 (8517960-8520596).

## 5. Conclusions

Activation of specific AGB1-NDL module interaction might be stress specific and hormone regulated, specificity of action is further added in different stages of growth and development due to differential expression patterns of *NDL* members. NDL1 protein stability in young primary root needs presence of AGB1, as NDL1 undergoes proteosomal degradation in absence of AGB1 [14]. Though AGB1 physically interacts with all NDLs it might differentially regulate their stability and hence their function during different stresses, effect of AGB1 on protein stability of NDL2 and NDL3 is still pending and needs to be characterized. Interestingly, Yeast 2 Hybrid (Y2H) confirmed NDL1 interactors includes-Annexin 1 (ANNAT1-has role in drought stress), Sodium and Lithium-Tolerant 1 (SLT1 involved in salt stress), Lesion Stimulating Disease 1 (LSD1 regulates cold stress), O-Acetylserine (Thiol) Lyase (OAS-TL) Isoform A1 (OASA1 role in cadmium tolerance) and *Arabidopsis* Ribosomal Protein S27 (ARS27A involved in genotoxic stress) [3]. These interactions speculate that NDL proteins might plays role in stress signaling in the form of multimeric complexes with these stress effectors and other G protein core components (RGS1 and AGB1) in stress specific manner.

**Figure 10:**
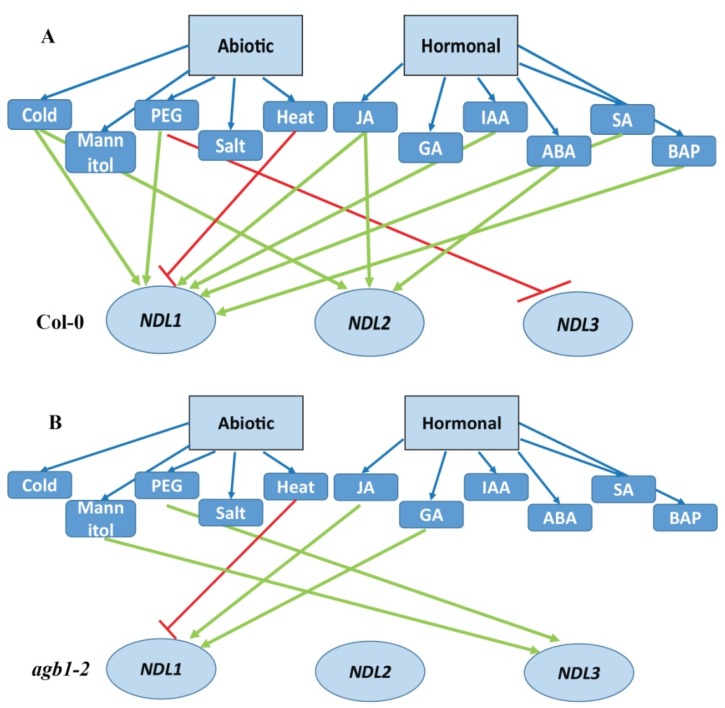
*AtNDLs* responses to various abiotic and hormonal treatments in presence and absence of AGB1: (**A**) Diagrammatic representation of *AtNDLs* in wild type background in response to various abiotic and hormonal treatments. (**B**) *AtNDLs* responses to various abiotic and hormonal treatments in *agb1-2* mutant background. The green lines indicates that the gene was up regulated while the red lines indicates the downregulation of gene after the respective treatments.

All of these findings together including regulatory elements in silico and in vivo expression profiling indicate that *NDL* family members along with *AGB1* play–key differential roles in different organs during different stages of plant growth and developmental. Collectively, our data suggest that *NDL-AGB1* modules are abiotic stress and hormone treatment-responsive and could be used as stress markers. In long run they could be potential candidates for crop improvement strategies (Figure 10).

## Figures and Tables

**Table 1:** List of common transcription factor (TF) binding sites in *NDL1*, *NDL2*, and *NDL3* promoter.

S.NO.	TFs	Function
1	MYB1AT	Dehydration-responsive elements
2	ARR1AT	Cytokinin response regulators
3	GT1CONSENSUS	Salicylic acid-responsive elements, Light-responsive elements
4	MYCCONSENSUSAT	dehydration-responsive
5	SURECOREATSULTR11	SURE contains auxin response factor (ARF) binding sequence
6	L1BOXATPDF1	MYB binding motif
7	WBOXATNPR1	Salicyclic acid-responsive elements
8	MYBCOREATCYCB1	Cyclin B1-responsive elements
9	ABRELATERD1	ABA-responsive elements
10	ANAERO1CONSENSUS	Anaerobic-responsive elements
11	WBBOXPCWRKY1	Pathogenesis-related elements
12	LEAFYATAG	Target sequence of LEAFY
13	P1BS	Phosphate starvation-responsive elements
14	SORLIP1AT	Light-responsive elements
15	MYB1LEPR	Defence-related elements

**Table 2:** List of specific TFs binding sites in promoter region of *NDL1*, *NDL2*, and *NDL3*.

S.NO.	TFs	Function
*NDL1*	E2FAT	Cell cycle-responsive elements
	RHERPATEXPA7	Root hair specific-cis elements
	SBOXATRBCS	Sugar and ABA-responsive elements
*NDL2*	ABREATCONSENSUS	ABA-responsive elements
	ABREATRD22	ABA-responsive elements
	ABRERATCAL	Ca^2+^-responsive elements
	ACGTABREMOTIFA2OSEM	ABA-responsive elements
	GADOWNAT	GA-responsive elements
	MYBATRD22	Dehydration-responsive elements
	SITEIIATCYTC	Responsible for oxidative phosphorylation
	T/GBOXATPIN2	JA-responsive elements
	UP1ATMSD	Upregulation after main stem decapitation
*NDL3*	ABRERATCAL	Ca^2+^-responsive elements
	AGL2ATCONSENSUS	AGAMOUS-LIKE 2
	CGCGBOXAT	Ca^2+^/Calmodulin response elements
	GCCCORE	Pathogen-responsive elements

**Table 3:** *AtNDLs* localization during various stages of development and after abiotic and hormonal treatments.

			Col-0			*agb1-2*		
Stage	Organ	Tissue	*AtNDL1*	*AtNDL2*	*AtNDL3*	*AtNDL1*	*AtNDL2*	*AtNDL3*
8-day old seedlings	PR	RT	**++**	**−**	**++**	**−**	**−**	**++**
		CDZ	**++**	**−**	**++**	**++**	**−**	**++**
		EZ	**++**	**−**	**−**	**++**	**−**	**−**
		MZ	**++**	**++**	**−**	**−**	**++**	**−**
	LR		**++**	**−**	**++**	**++**	**−**	**++**
	Hypocotyl		**−**	**++**	**−**	**−**	**++**	**−**
	Cotyledons		**++**	**++**	**−**	**−**	**++**	**−**
	Leaves		**++**	**++**	**++**	**++**	**++**	**++**
6-day old seedlings (Cold)	PR	RT	**++**	**−**	**++**	**−**	**−**	**++**
		CDZ	**++**	**−**	**++**	**++**	**−**	**++**
		EZ	**++**	**−**	**−**	**++**	**−**	**−**
		MZ	**+**	**+**	**−**	**+**	**++**	**−**
	Hypocotyl		**++**	**++**	**++**	**−**	**++**	**−**
	Cotyledons		**+++**	**+++**	**++**	**−**	**++**	**−**
6-day old seedlings (Heat)	PR	RT	**−**	**−**	**++**	**−**	**−**	**++**
		CDZ	**−**	**−**	**++**	**−**	**−**	**++**
		EZ	**−**	**−**	**−**	**−**	**−**	**−**
		MZ	**−**	**++**	**−**	**−**	**++**	**−**
	Hypocotyl		**+**	**++**	**−**	**−**	**++**	**−**
	Cotyledons		**−**	**++**	**+**	**−**	**++**	**−**
6-day old seedlings (Mannitol)	PR	RT	**++**	**−**	**++**	**−**	**−**	**++**
		CDZ	**+**	**−**	**+**	**++**	**−**	**++**
		EZ	**+**	**−**	**−**	**−**	**−**	**−**
		MZ	**−**	**++**	**−**	**−**	**++**	**−**
	Hypocotyl		**++**	**++**	**−**	**−**	**++**	**−**
	Cotyledons		**++**	**++**	**−**	**+**	**++**	**+**
6-day old seedlings (PEG)	PR	RT	**+++**	**−**	**−**	**−**	**−**	**+++**
		CDZ	**+++**	**−**	**−**	**+++**	**−**	**++**
		EZ	**++**	**−**	**−**	**+**	**−**	**−**
		MZ	**−**	**++**	**−**	**−**	**++**	**−**
	Hypocotyl		**−**	**++**	**+**	**−**	**++**	**−**
	Cotyledons		**++**	**++**	**+**	**−**	**++**	**−**
6-day old seedlings (Salt)	PR	RT	**++**	**−**	**++**	**−**	**−**	**++**
		CDZ	**++**	**−**	**++**	**++**	**−**	**++**
		EZ	**+**	**−**	**−**	**++**	**−**	**−**
		MZ	**+**	**++**	**−**	**−**	**++**	**−**
	Hypocotyl		**++**	**++**	**+**	**−**	**++**	**+**
	Cotyledons		**++**	**++**	**+**	**−**	**++**	**−**
6-day old seedlings (ABA)	PR	RT	**++**	**−**	**++**	**−**	**−**	**++**
		CDZ	**++**	**−**	**++**	**++**	**−**	**++**
		EZ	**++**	**−**	**−**	**++**	**−**	**−**
		MZ	**−**	**++**	**−**	**++**	**++**	**−**
	Hypocotyl		**−**	**++**	**−**	**−**	**++**	**−**
	Cotyledons		**++**	**+++**	**++**	**−**	**++**	**−**
6-day old seedlings (GA)	PR	RT	**++**	**−**	**++**	**−**	**−**	**++**
		CDZ	**++**	**−**	**++**	**++**	**−**	**++**
		EZ	**++**	**−**	**−**	**+++**	**−**	**−**
		MZ	**+**	**+**	**−**	**++**	**++**	**−**
	Hypocotyl		**+**	**++**	**+**	**−**	**++**	**−**
	Cotyledons		**++**	**++**	**+**	**−**	**++**	**−**
6-day old seedlings (IAA)	PR	RT	**++**	**−**	**++**	**−**	**−**	**++**
		CDZ	**+++**	**−**	**++**	**++**	**−**	**++**
		EZ	**+++**	**−**	**−**	**++**	**−**	**−**
		MZ	**++**	**+**	**−**	**+**	**++**	**−**
	Hypocotyl		**++**	**++**	**+**	**−**	**++**	**−**
	Cotyledons		**++**	**++**	**++**	**−**	**++**	**−**
6-day old seedlings (JA)	PR	RT	**++**	**−**	**++**	**+++**	**−**	**++**
		CDZ	**++**	**−**	**++**	**+++**	**−**	**++**
		EZ	**++**	**−**	**−**	**+++**	**−**	**−**
		MZ	**++**	**+**	**−**	**++**	**++**	**−**
	Hypocotyl		**++**	**++**	**−**	**−**	**++**	**−**
	Cotyledons		**++**	**+++**	**−**	**−**	**++**	**−**
6-day old seedlings (SA)	PR	RT	**++**	**−**	**++**	**−**	**−**	**++**
		CDZ	**++**	**−**	**++**	**+**	**−**	**++**
		EZ	**+**	**−**	**−**	**+**	**−**	**−**
		MZ	**−**	**++**	**−**	**−**	**++**	**−**
	Hypocotyl		**+**	**++**	**+**	**−**	**++**	**−**
	Cotyledons		**++**	**++**	**+**	**−**	**++**	**+**
6-day old seedlings (BAP)	PR	RT	**++**	**−**	**++**	**−**	**−**	**++**
		CDZ	**++**	**−**	**++**	**++**	**−**	**++**
		EZ	**++**	**−**	**−**	**++**	**−**	**−**
		MZ	**+**	**++**	**−**	**++**	**++**	**−**
	Hypocotyl		**−**	**++**	**−**	**−**	**++**	**−**
	Cotyledons		**+++**	**++**	**+**	**−**	**++**	**+**
6-day old seedlings (Control)	PR	RT	**++**	**−**	**++**	**−**	**−**	**++**
		CDZ	**++**	**−**	**++**	**++**	**−**	**++**
		EZ	**++**	**−**	**−**	**++**	**−**	**−**
		MZ	**−**	**++**	**−**	**++**	**++**	**−**
	Hypocotyl		**−**	**++**	**++**	**−**	**++**	**−**
	Cotyledons		**++**	**++**	**++**	**−**	**++**	**−**

PR = Primary Root, LR = Lateral Root, CDZ = Cell division Zone, EZ = Elongation Zone, MZ = Maturation Zone, Root tip = RT. (-) No GUS staining, (++) basal staining level in each genotype without any treatment, (+++) increased level of staining, (+) decreased levels of staining compared to basal genotypic levels.

## Supplementary Materials

Can be found at https://www.mdpi.com/1422-0067/20/19/4736/s1.

## References

[1] Colaneri A.C., Tunc-Ozdemir M., Huang J.P., Jones A.M. (2014). Growth attenuation under saline stress is mediated by the heterotrimeric G protein complex. BMC Plant Biol..

[2] Ming C.H., Xu D.B., Fang G.N., Wang E.H., Gao S.Q., Xu Z.S., Li L.C., Zhang X.H., Miin D.H. (2015). G-protein β subunit AGB1 positively regulates salt stress tolerance in *Arabidopsis*. J. Integr. Agric..

[3] Klopffleisch K., Phan N., Augustin K., Bayne R.S., Booker K.S., Botella J.R., Carpita N.C., Carr T., Chen J.G., Cooke T.R. (2011). *Arabidopsis* G-protein interactome reveals connections to cell wall carbohydrates and morphogenesis. Mol. Syst. Biol..

[4] Kalaydjieva L., Gresham D., Gooding R., Heather L., Baas F., De Jonge R., Blechschmidt K., Angelicheva D., Chandler D., Worsley P. (2000). N-myc downstream-regulated gene 1 is mutated in hereditary motor and sensory neuropathy–Lom. Am. J. Hum. Genet..

[5] Yan S.F., Lu J., Zou Y.S., Soh-Won J., Cohen D.M., Buttrick P.M., Cooper D.R., Steinberg S.F., Mackman N., Pinsky D.J. (1999). Hypoxia-associated induction of early growth response-1 gene expression. J. Biol. Chem..

[6] Salnikow K., Su W., Blagosklonny M.V., Costa M. (2000). Carcinogenic metals induce hypoxia-inducible factor-stimulated transcription by reactive oxygen species-independent mechanism. Cancer Res..

[7] Lachat P., Shaw P., Gebhard S., Van Belzen N., Chaubert P., Bosman F.T. (2002). Expression of NDRG1, a differentiation-related gene, in human tissues. Histochem. Cell Biol..

[8] Salnikow K., Kluz T., Costa M., Piquemal D., Demidenko Z.N., Xie K., Blagosklonny M.V. (2002). The regulation of hypoxic genes by calcium involves c-Jun/AP-1, which cooperates with hypoxia-inducible factor 1 in response to hypoxia. Mol. Cell. Boil..

[9] Wu D., Zhau H.E., Huang W.C., Iqbal S., Habib F.K., Sartor O., Cvitanovic L., Marshall F.F., Xu Z., Chung L.W.K. (2007). cAMP-responsive element-binding protein regulates vascular endothelial growth factor expression: Implication in human prostate cancer bone metastasis. Oncogene.

[10] Zhou D., Salnikow K., Costa M. (1998). Cap43, a novel gene specifically induced by Ni^2+^ compounds. Cancer Res..

[11] Krauter-Canham R., Bronner R., Evrard J.L., Hahne G., Friedt W., Steinmetz A. (1997). A transmitting tissue-and pollen-expressed protein from sunflower with sequence similarity to the human RTP protein. Plant Sci..

[12] Lazarescu E., Friedt W., Horn R., Steinmetz A. (2006). Expression analysis of the sunflower SF21 gene family reveals multiple alternative and organ-specific splicing of transcripts. Gene.

[13] Lazarescu E., Friedt W., Steinmetz A. (2010). Organ-specific alternatively spliced transcript isoforms of the sunflower *SF21C* gene. Plant Cell Rep..

[14] Mudgil Y., Uhrig J.F., Zhou J., Temple B., Jiang K., Jones A.M. (2009). *Arabidopsis N-MYC DOWNREGULATED-LIKE1*, a positive regulator of auxin transport in a G protein–mediated pathway. Plant Cell..

[15] Rymaszewski W., Vile D., Bediee A., Dauzat M., Luchaire N., Kamrowska D., Granier C., Hennig J. (2017). Stress-related gene expression reflects morphophysiological responses to water deficit. Plant Physiol..

[16] Mudgil Y., Ghawana S., Jones A.M. (2013). N-MYC down-regulated-like proteins regulate meristem initiation by modulating auxin transport and MAX2 expression. PLoS ONE.

[17] Luscher B., Eisenman R.N. (1990). New light on Myc and Myb. Part II. Myb. Genes Dev..

[18] Abe H., Urao T., Ito T., Seki M., Shinozaki K., Yamaguchi-Shinozaki K. (2003). *Arabidopsis AtMYC2* (bHLH) and *AtMYB2* (MYB) function as transcriptional activators in abscisic acid signaling. Plant Cell..

[19] Yamaguchi-Shinozaki K., Shinozaki K. (2005). Organization of *cis*-acting regulatory elements in osmotic-and cold-stress-responsive promoters. Trends Plant Sci..

[20] Simpson S.D., Nakashima K., Narusaka Y., Seki M., Shinozaki K., Yamaguchi-Shinozaki K. (2003). Two different novel *cis*-acting elements of *erd1*, a *clpA* homologous *Arabidopsis* gene function in induction by dehydration stress and dark-induced senescence. Plant J..

[21] Du H., Huang F., Wu N., Li X., Hu H., Xiong L. (2018). Integrative regulation of drought escape through ABA-dependent and-independent pathways in rice. Mol. Plant..

[22] Chinnusamy V., Ohta M., Kanrar S., Lee B.H., Hong X., Agarwal M., Zhu J.K. (2003). ICE1: A regulator of cold-induced transcriptome and freezing tolerance in *Arabidopsis*. Genes Dev..

[23] Wang C., Wang Y., Pan Q., Chen S., Feng C., Hai J., Li H. (2019). Comparison of Trihelix transcription factors between wheat and *Brachypodiumdistachyon* at genome-wide. BMC Genom..

[24] Rouached H., Secco D., Arpat B., Poirier Y. (2011). The transcription factor PHR1 plays a key role in the regulation of sulfate shoot-to-root flux upon phosphate starvation in *Arabidopsis*. BMC Plant Biol..

[25] Sobkowiak L., Bielewicz D., Małecka E., Jakobsen I., Albrechtsen M., Szweykowska-Kulinska Z., Pacak A.M. (2012). The role of the P1BS element containing promoter-driven genes in Pi transport and homeostasis in plants. Front. Plant Sci..

[26] Helin K. (1998). Regulation of cell proliferation by the E2F transcription factors. Curr. Opin. Genet. Dev..

[27] Lammens T., Li J., Leone G., De Veylder L. (2009). Atypical E2Fs: New players in the E2F transcription factor family. Trends Cell Biol..

[28] De Veylder L., Beeckman T., Inze D. (2007). The ins and outs of the plant cell cycle. Nat. Rev. Mol. Cell Biol..

[29] Narusaka Y., Nakashima K., Shinwari Z.K., Sakuma Y., Furihata T., Abe H., Narusaka M., Shinozaki K., Yamaguchi-Shinozaki K. (2003). Interaction between two cis-acting elements, ABRE and DRE, in ABA-dependent expression of *Arabidopsis* rd29A gene in response to dehydration and high-salinity stresses. Plant J..

[30] Hattori T., Totsuka M., Hobo T., Kagaya Y., Yamamoto-Toyoda A. (2002). Experimentally determined sequence requirement of ACGT-containing abscisic acid response element. Plant Cell Physiol..

[31] Yang X., Yang Y.N., Xue L.J., Zou M.J., Liu J.Y., Chen F., Xue H.W. (2011). Rice ABI5-Like1 regulates abscisic acid and auxin responses by affecting the expression of ABRE-containing genes. Plant Physiol..

[32] Nakabayashi K., Okamoto M., Koshiba T., Kamiya Y., Nambara E. (2005). Genome-wide profiling of stored mRNA in *Arabidopsis thaliana* seed germination: Epigenetic and genetic regulation of transcription in seed. Plant J..

[33] Skriver K., Olsen F.L., Rogers J.C., Mundy J. (1991). *Cis*-acting DNA elements responsive to gibberellin and its antagonist abscisic acid. Proc. Natl. Acad. Sci. USA.

[34] Ogawa M., Hanada A., Yamauchi Y., Kuwahara A., Kamiya Y., Yamaguchi S. (2003). Gibberellin biosynthesis and response during *Arabidopsis* seed germination. Plant Cell..

[35] Boter M., Ruiz-Rivero O., Abdeen A., Prat S. (2004). Conserved MYC transcription factors play a key role in jasmonate signaling both in tomato and *Arabidopsis*. Genes Dev..

[36] Kaplan B., Davydov O., Knight H., Galon Y., Knight M.R., Fluhr R., Fromm H. (2006). Rapid transcriptome changes induced by cytosolic Ca^2+^ transients reveal ABRE-related sequences as Ca^2+^-responsive *cis*-elements in *Arabidopsis*. Plant Cell..

[37] Yang T., Poovaiah B.W. (2002). A calmodulin-binding/CGCG box DNA-binding protein family involved in multiple signaling pathways in plants. J. Biol. Chem..

[38] Du L., Ali G.S., Simons K.A., Hou J., Yang T., Reddy A.S., Poovaiah B.W. (2009). Ca^2+^/calmodulin regulates salicylic-acid-mediated plant immunity. Nature.

[39] Brown R.L., Kazan K., McGrath K.C., Maclean D.J., Manners J.M.A. (2003). Role for the GCC-box in jasmonate-mediated activation of the *PDF1.2* gene of *Arabidopsis*. Plant Physiol..

[40] Yang J.H., Wang H. (2016). Molecular mechanisms for vascular development and secondary cell wall formation. Front. Plant Sci..

[41] Huang T., Harrar Y., Lin C., Reinhart B., Newell N.R., Talavera-Rauh F., Hokin S.A., Barton M.K., Kerstetter R.A. (2014). *Arabidopsis* KANADI1 acts as a transcriptional repressor by interacting with a specific *cis*-element and regulates auxin biosynthesis, transport, and signaling in opposition to HD-ZIPIII factors. Plant Cell..

[42] Emery J.F., Floyd S.K., Alvarez J., Eshed Y., Hawker N.P., Izhaki A., Baum S.F., Bowman J.L. (2003). Radial patterning of *Arabidopsis* shoots by class III HD-ZIP and KANADI genes. Curr. Biol..

[43] Izhaki A., Bowman J.L. (2007). KANADI and class III HD-Zip gene families regulate embryo patterning and modulate auxin flow during embryogenesis in *Arabidopsis*. Plant Cell..

[44] Footitt S., Douterelo-Soler I., Clay H., Finch-Savage W.E. (2011). Dormancy cycling in *Arabidopsis* seeds is controlled by seasonally distinct hormone-signaling pathways. Proc. Natl. Acad. Sci. USA.

[45] Xu D.B., Chen M., Ma Y.N., Xu Z.S., Li L.C., Chen Y.F., Ma Y.Z.A. (2015). G-protein β subunit, AGB1, negatively regulates the ABA response and drought tolerance by down-regulating AtMPK6-related pathway in *Arabidopsis*. PLoS ONE.

[46] Clough S.J., Bent A.F. (1998). Floral dip: A simplified method for *Agrobacterium*-mediated transformation of *Arabidopsis thaliana*. Plant J..

[47] Doyle J.J., Doyle J.L. (1990). Isolation of plant DNA from fresh tissue. Focus.

[48] Chang W.C., Lee T.Y., Huang H.D., Huang H.Y., Pan R.L. (2008). PlantPAN: Plant promoter analysis navigator, for identifying combinatorial *cis*-regulatory elements with distance constraint in plant gene groups. BMC Genom..

[49] Jefferson R.A., Kavanagh T.A., Bevan M.W. (1987). GUS fusions: Beta-glucuronidase as a sensitive and versatile gene fusion marker in higher plants. EMBO J..

[50] Bradford M.M. (1976). ARapid and sensitive method for the quantitation of microgram quantities of protein utilizing the principle of protein-dye binding. Anal. Biochem..

[51] Ren Y., Zhao J. (2009). Functional analysis of the rice metallothionein gene OsMT2b promoter in transgenic *Arabidopsis* plants and rice germinated embryos. Plant Sci..

