# Supplementary material for: Arabidopsis NDL-AGB1 modules Play Role in Abiotic Stress and Hormonal Responses Along with Their Specific Functions
Source: Int J Mol Sci. 2019 Sep 24;20(19):4736. doi: 10.3390/ijms20194736 (PMC6801982; doi:10.3390/ijms20194736)
Supplement: Supplementary file 1 [file ijms-20-04736-s001.zip › Supp material/supp material Revised Final.pdf]

Supplementary Material

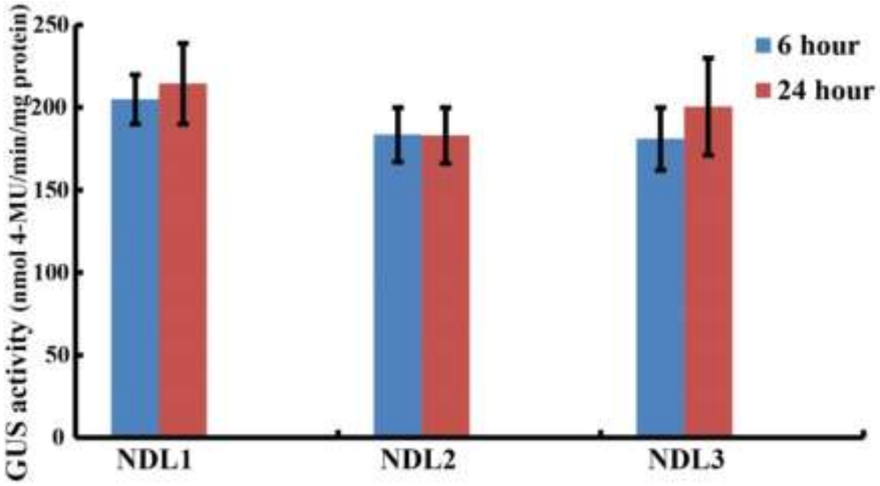

Supplementary Figure 1: ***NDLs* promoter-GUS/β-glucuronidase activity was tested using MUG assay.** MUG assay was done for all the three *NDL* members in Col-0 background using six day old seedlings after putting them in plain liquid ½ MS for two time points (6 hours and 24 hours). Result shown is representative of two independent biological replicates (n = 10 seedlings in each experiment). No significant difference was observed between genotypes and time points.

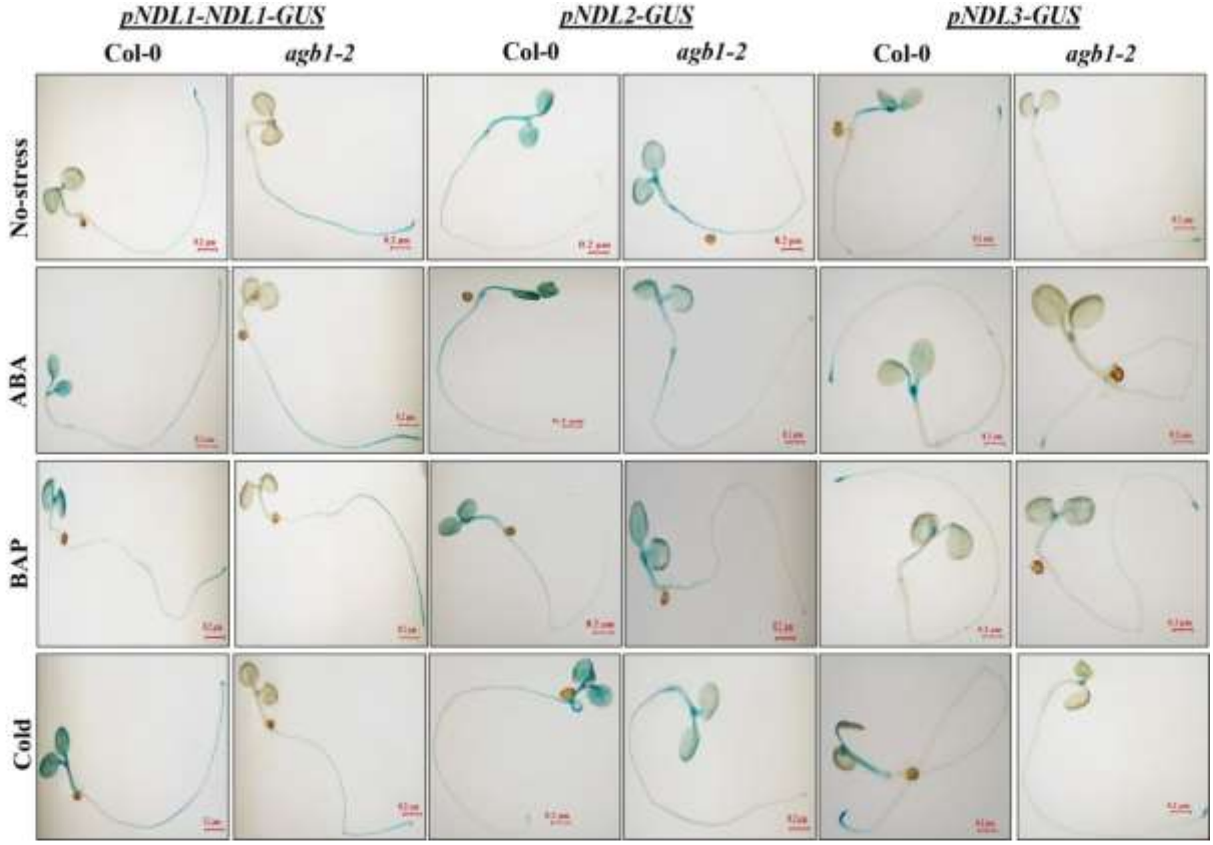

Supplementary Figure 2. Histochemical GUS analysis of all three *NDL* family members in wild type Col-0 and *agb1-2* mutant background, in response to ABA, BAP and cold stress. GUS staining was done in seedlings that were six days old and 24 hours of stress treatment was given. Result shown is representative of three independent biological replicates (n ≥ 10 in each experiment). Scale Bar=0.2µM

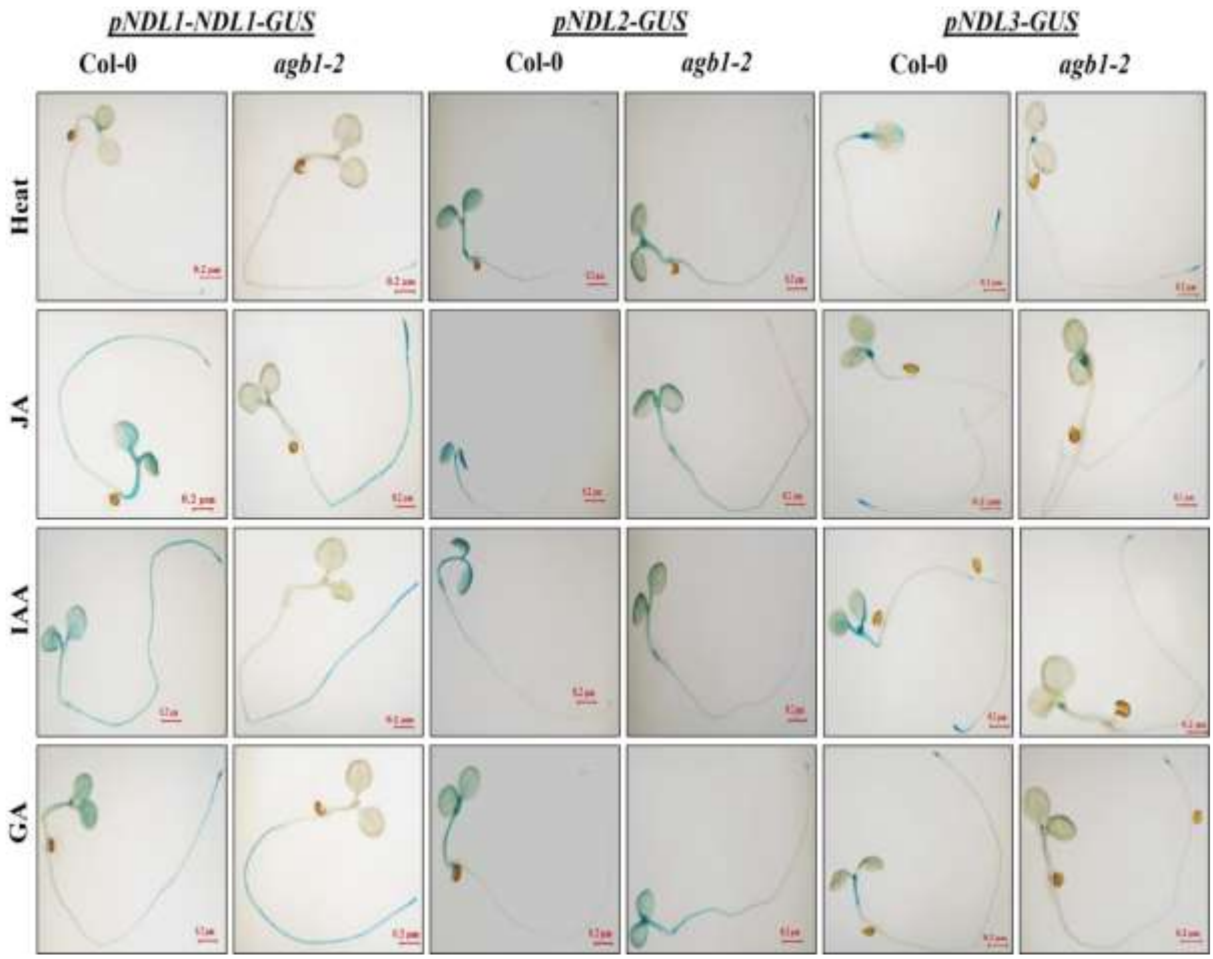

Supplementary Figure 3. Histochemical GUS analysis of all three *NDL* family members in wild type Col-0 and *agb1-2* mutant background , in response to JA, IAA and GA treatment. GUS staining was done in seedlings that were six days old and 24 hours of stress treatment was given Result shown is representative of three independent biological replicates (n ≥ 10 in each experiment). Scale Bar=0.2μM

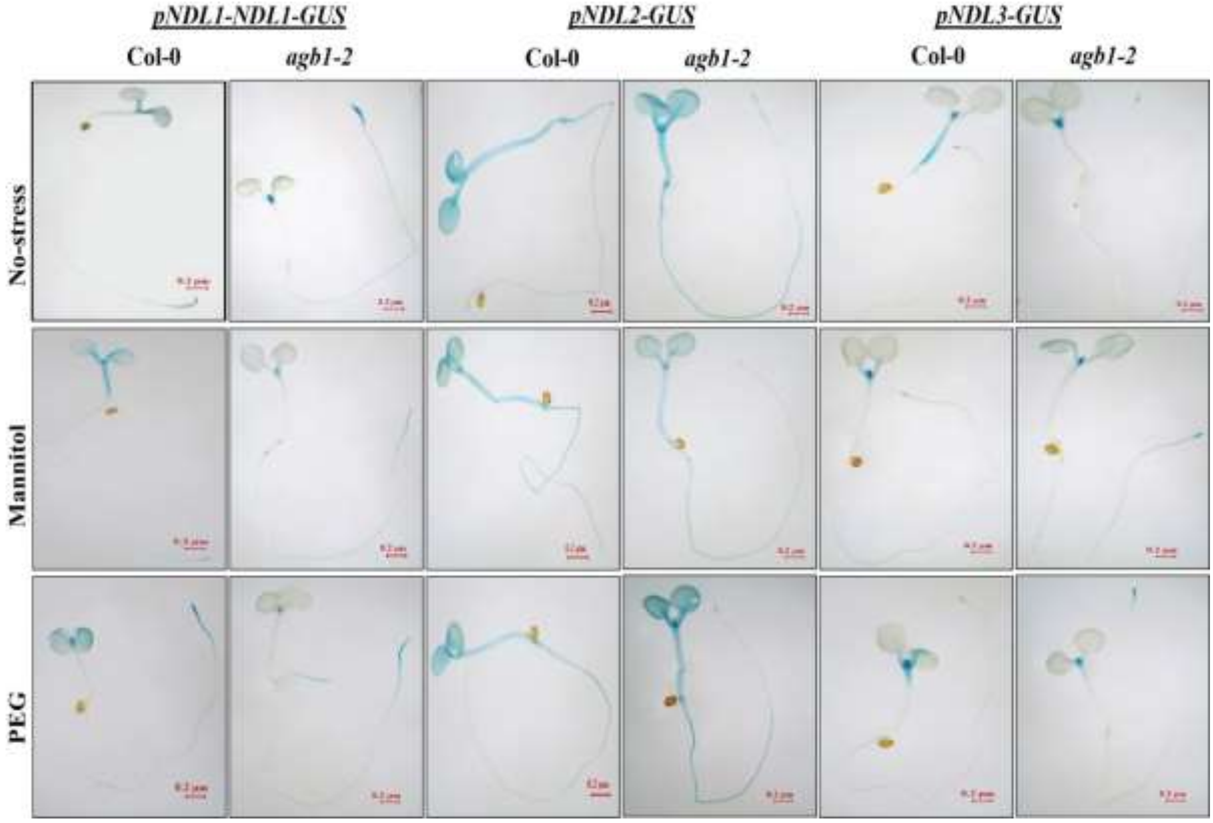

Supplementary Figure 4. Histochemical GUS analysis of all three *NDL* family members in wild type Col-0 and *agb1-2* mutant background , in response to Mannitol and PEG treatment. GUS staining was done in seedlings that were six days old and 24 hours of stress treatment was given. Result shown is representative of three independent biological replicates (n ≥ 10 in each experiment). Scale Bar=0.2μM

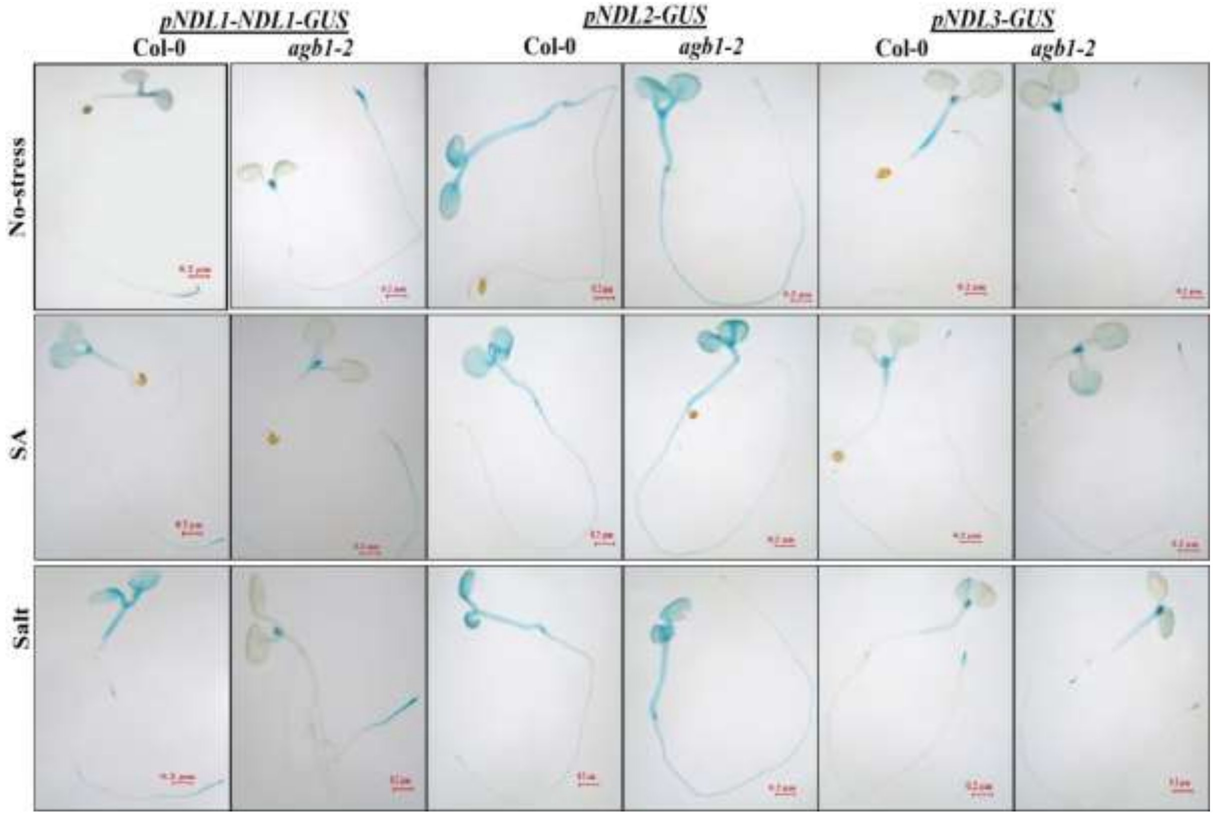

Supplementary Figure 5. Histochemical GUS analysis of all three *NDL* family members in wild type Col-0 and and *agb1 - 2* mutant background , in response to SA and NaCl treatment. GUS staining was done in seedlings that were six days old and 24 hours of stress treatment was given. Result shown is representative of three independent biological replicates (n ≥ 10 in each experiment). Scale Bar=0.2µM

Supplementary.Table 1. Primers used in the study

**Accession number**	**Gene Name**	**Amplicon**	**Forward primer**	**Reverse primer**
AT5G56750	*NDL1* full length	3393bp	*NDL1* promoter sp. 5′-CACCTCTGATGGTTTAAGATTAGTCCATTTCT-3′	*NDL1* gene sp. 5′-CTATAGAGCGAGTCGTGTCT-3′
AT5G11790	*NDL2* promoter	1386bp	*NDL2* promoter sp. 5′-CACCCTCATCTAATTGGGA-3′ ′	*NDL2* promoter sp. 5′-GCCATCTCCTTCTCTCTCT-3′
AT2G19620	*NDL3* promoter	1280bp	*NDL3* promoter sp. 5′-CACCTTAGCCATAAAATTGAC-3'	*NDL3* promoter sp. 5′-GCATACAAACTAAAATCAAGAACAC-3'
