# Supplementary material for: Arabidopsis NDL-AGB1 modules Play Role in Abiotic Stress and Hormonal Responses Along with Their Specific Functions
Source: Int J Mol Sci. 2019 Sep 24;20(19):4736. doi: 10.3390/ijms20194736 (PMC6801982; doi:10.3390/ijms20194736)
Supplement: Supplementary file 1 [file ijms-20-04736-s001.zip › Supp material/supp table 1.docx]

**Supplementary.Table S1. Primers used in the study.**

| **Accession number** | **Gene Name** | **Amplicon** | **Forward Primer** | **Reverse Primer** |
| --- | --- | --- | --- | --- |
| AT5G56750 | *NDL1* full length | 3393bp | *NDL1* promoter sp. 5´-CACCTCTGATGGTTTAAGATTAGTCCATTTCT-3´ | *NDL1* gene sp. 5´-CTATAGAGCGAGTCGTGTCT-3´ |
| AT5G11790 | *NDL2* promoter | 1386bp | *NDL2* promoter sp. 5´-CACC CTCATCTAATTGGGA-3´ ´ | *NDL2* promoter sp. 5´-GCCATCTCCTTCTCTCTCT-3´ |
| AT2G19620 | *NDL3* promoter | 1280bp | *NDL3* promoter sp. 5´-CACCTTAGCCATAAAATTGAC-3' | *NDL3* promoter sp. 5´-GCATACAAACTAAAATCAAGAACAC-3' |
