# Supplementary figures and images for: Arabidopsis NDL-AGB1 modules Play Role in Abiotic Stress and Hormonal Responses Along with Their Specific Functions
Source: Int J Mol Sci. 2019 Sep 24;20(19):4736. doi: 10.3390/ijms20194736 (PMC6801982; doi:10.3390/ijms20194736)

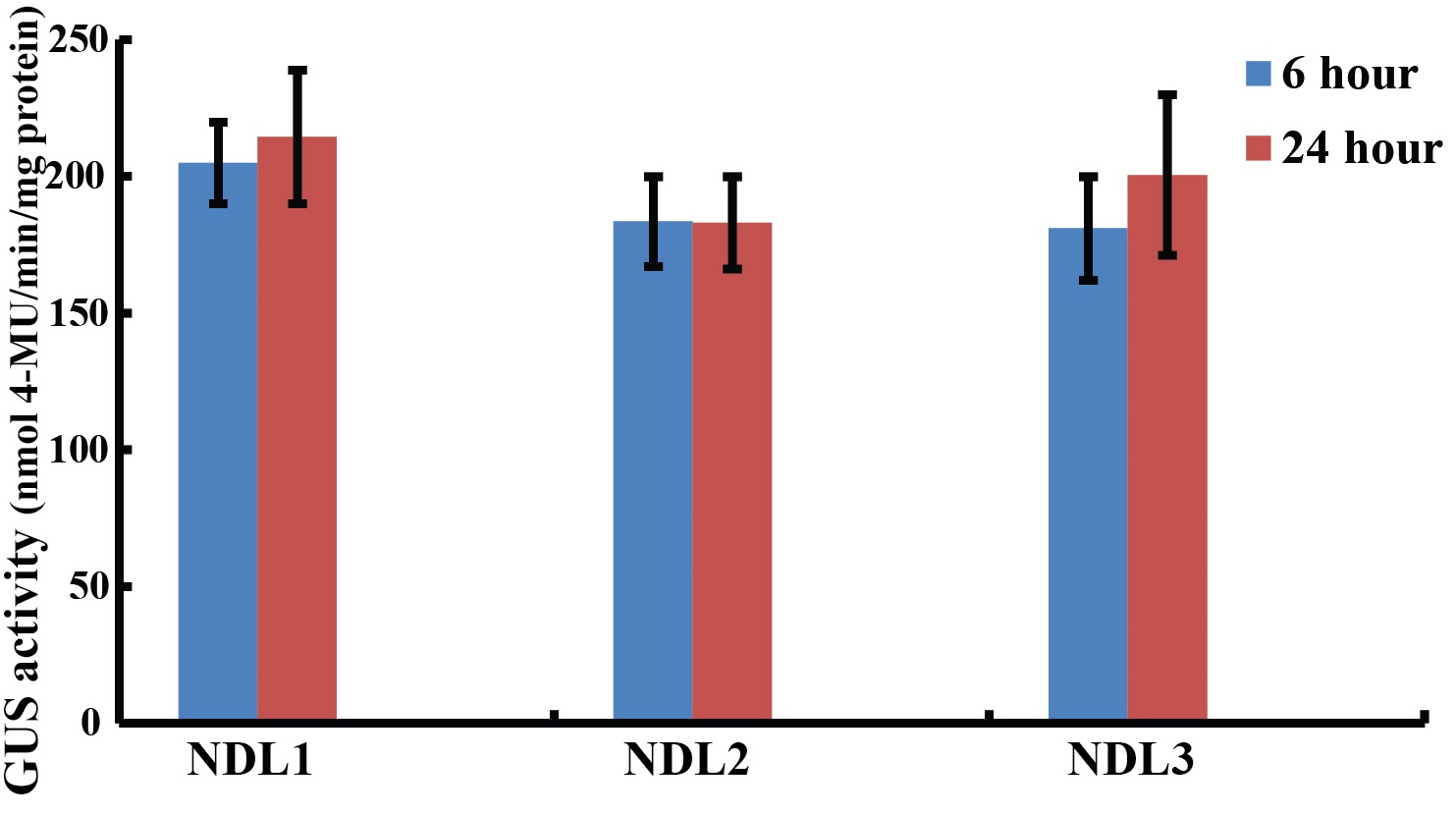

Supplement: Supplementary file 1 [file ijms-20-04736-s001.zip › Supp material/supp fig1.jpg]

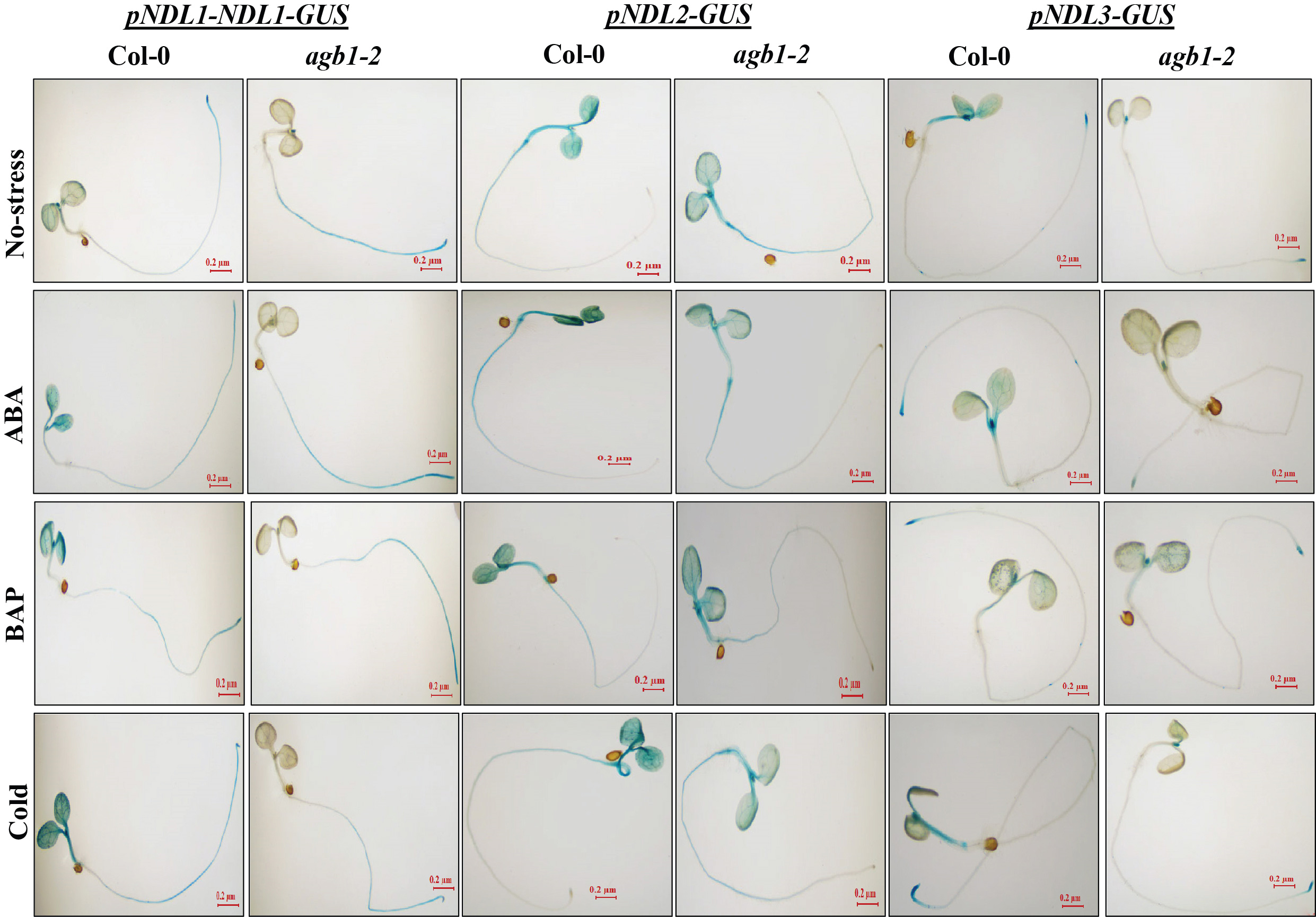

Supplement: Supplementary file 1 [file ijms-20-04736-s001.zip › Supp material/supp fig2.jpg]

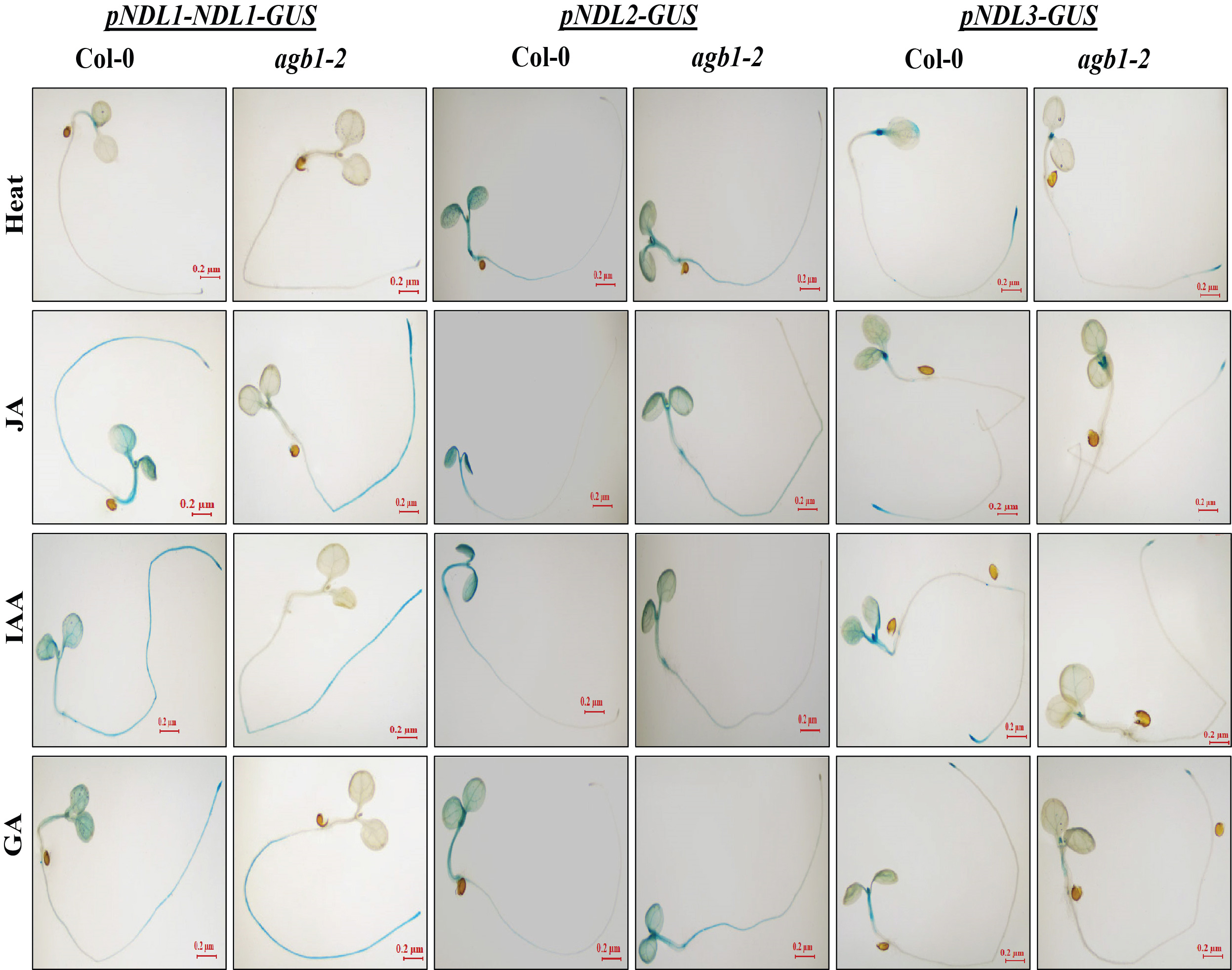

Supplement: Supplementary file 1 [file ijms-20-04736-s001.zip › Supp material/supp fig3.jpg]

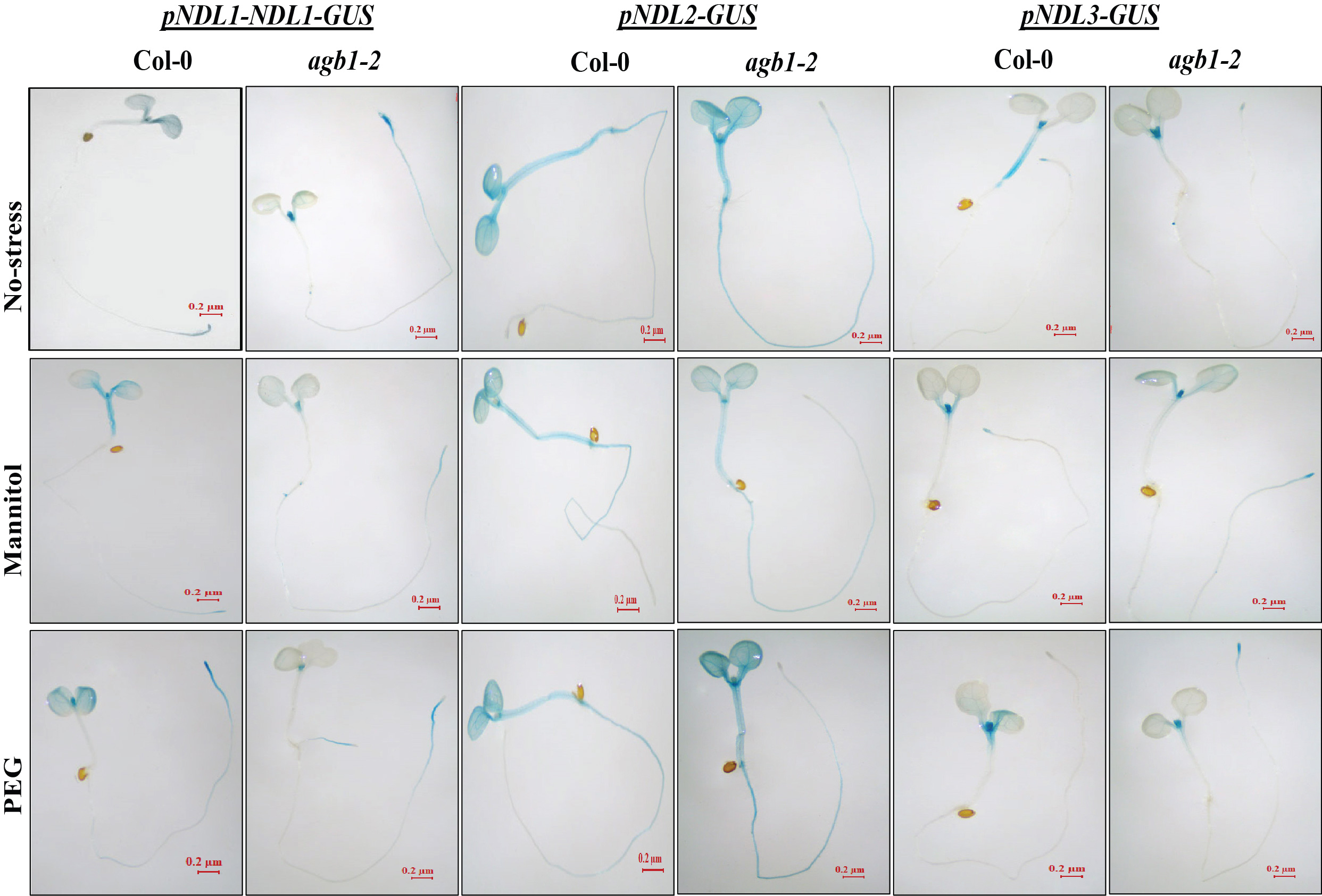

Supplement: Supplementary file 1 [file ijms-20-04736-s001.zip › Supp material/supp fig4.jpg]

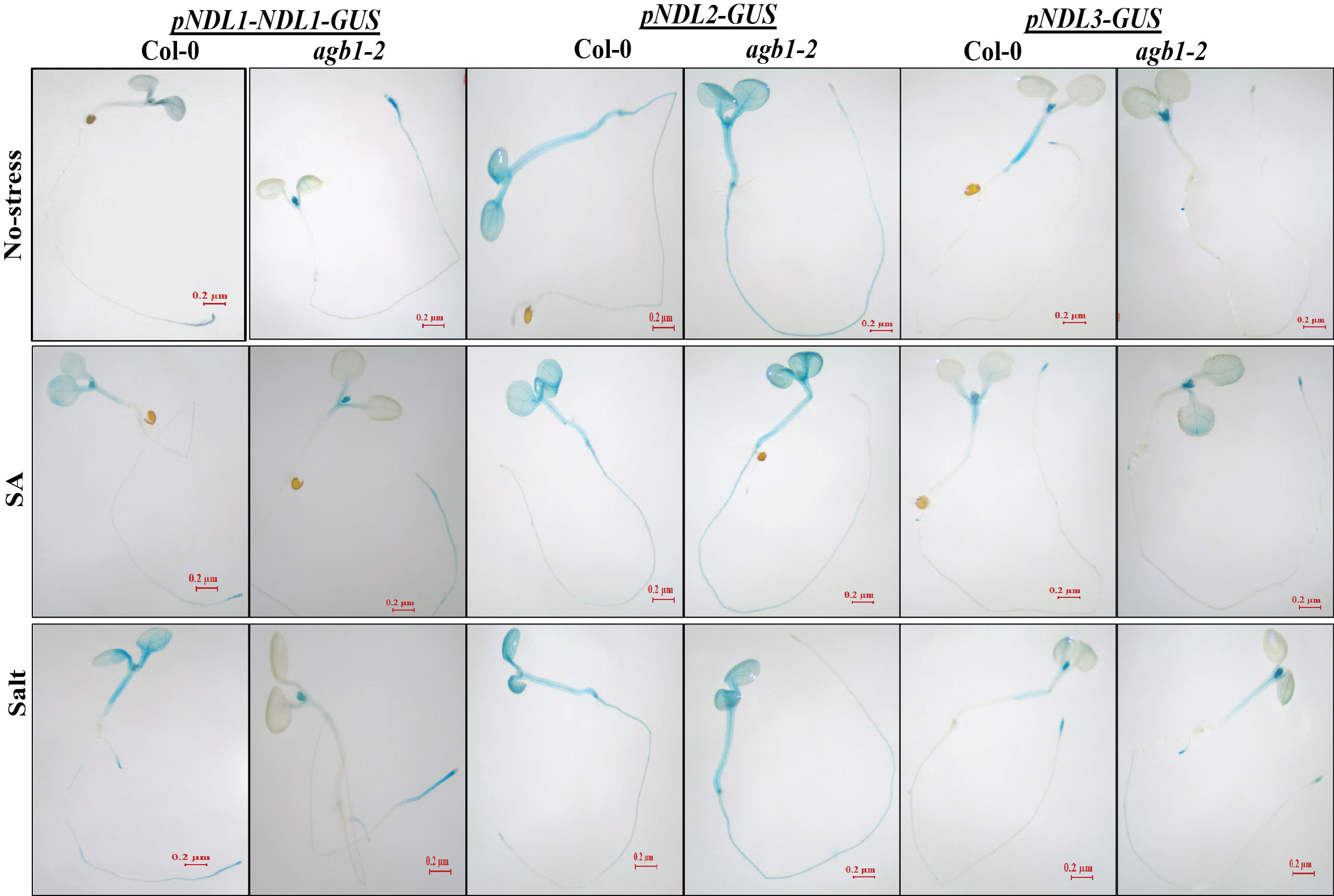

Supplement: Supplementary file 1 [file ijms-20-04736-s001.zip › Supp material/supp fig5.jpg]
